# Increased Inlet Blood Flow Velocity Predicts Low Wall Shear Stress in the Cephalic Arch of Patients with Brachiocephalic Fistula Access

**DOI:** 10.1371/journal.pone.0152873

**Published:** 2016-04-13

**Authors:** Mary Hammes, Michael Boghosian, Kevin Cassel, Sydeaka Watson, Brian Funaki, Taral Doshi, S. M. Javid Mahmoudzadeh Akherat, Jane Hines, Fredric Coe

**Affiliations:** 1 Department of Medicine, Nephrology Section, The University of Chicago, Chicago, IL, United States of America; 2 Department of Mechanical, Materials and Aerospace Engineering, Illinois Institute of Technology, Chicago, IL, United States of America; 3 Department of Public Health Sciences, Biostatistics Laboratory, The University of Chicago, Chicago, IL, United States of America; 4 Department of Radiology, The University of Chicago, Chicago, IL, United States of America; Technion - Israel Institute of Technology, ISRAEL

## Abstract

**Background:**

An autogenous arteriovenous fistula is the optimal vascular access for hemodialysis. In the case of brachiocephalic fistula, cephalic arch stenosis commonly develops leading to access failure. We have hypothesized that a contribution to fistula failure is low wall shear stress resulting from post-fistula creation hemodynamic changes that occur in the cephalic arch.

**Methods:**

Twenty-two subjects with advanced renal failure had brachiocephalic fistulae placed. The following procedures were performed at mapping (pre-operative) and at fistula maturation (8–32 weeks post-operative): venogram, Doppler to measure venous blood flow velocity, and whole blood viscosity. Geometric and computational modeling was performed to determine wall shear stress and other geometric parameters. The relationship between hemodynamic parameters and clinical findings was examined using univariate analysis and linear regression.

**Results:**

The percent low wall shear stress was linearly related to the increase in blood flow velocity (*p* < 0.01). This relationship was more significant in non-diabetic patients (*p* < 0.01) than diabetic patients. The change in global measures of arch curvature and asymmetry also evolve with time to maturation (*p* < 0.05).

**Conclusions:**

The curvature and hemodynamic changes during fistula maturation increase the percentage of low wall shear stress regions within the cephalic arch. Low wall shear stress may contribute to subsequent neointimal hyperplasia and resultant cephalic arch stenosis. If this hypothesis remains tenable with further studies, ways of protecting the arch through control of blood flow velocity may need to be developed.

## Introduction

Primary brachiocephalic fistulae (BCF) for hemodialysis frequently lose patency because stenosis occurs in the cephalic arch, a vein segment that connects the cephalic to the axillary vein [1, 2]. Stenosis is commonly ascribed to an increased blood flow rate, external musculoskeletal compression, or hypertrophy of valves [3,4]. We have considered the alternative hypothesis that high blood flows develop in the cephalic vein due to fistula creation and lead to local regions with abnormally low wall shear stress (WSS) in curved segments such as the cephalic arch. Low WSS is known to promote neointimal hyperplasia (NH) [5], which would narrow the vein lumen, potentially further disrupting flow dynamics, and lead to cephalic arch stenosis (CAS). Elsewhere [6], we have shown that the normally smooth curve of the arch can become more sharply angulated near its termination at the axillary vein, and that this occurs over an average interval of two to three years after fistula creation. More acute curvature is prone to disrupt smooth laminar flow and promote more complex behavior [7], such as separation [8], and exacerbate the likelihood of NH and stenosis [9].

There are many factors that may affect blood flow velocity (BFV) and adequate arteriovenous fistula (AVF) maturation in end-stage renal disease (ESRD) patients. Maturation is a process by which the vein undergoes arterialization, permitting it to withstand blood flows of 350–450 mL/min needed for hemodialysis [10]. Factors that influence maturation include age, history of vascular disease, diabetes, gender, race, underlying vein histology, and vein and artery diameters [10,11,12]. It is also important to note that the blood flow through the cephalic vein becomes pulsatile after creation of the fistula and maturation. Defective remodeling occurs as a consequence of diabetic arterial vascular disease [13], but it is unclear if this occurs in veins. It may not, because CAS appears less common in diabetics as compared to non-diabetics [14,15,16]. To date, outcomes for BCF are less than optimal. Once CAS becomes clinically evident, treatment options are costly and often fail [6]. Early studies in the maturation process of an AVF are necessary to understand the events that lead to CAS in the hope of prevention altogether.

Our purposes here were to quantify the geometric and hemodynamic characteristics of the cephalic arch during the maturation process and test four hypotheses concerning the mechanisms leading to CAS: 1) The geometric and hemodynamic changes that occur during maturation lead to regions of low WSS in the cephalic arch; 2) Low WSS is directly related to increased blood flow velocity (BFV) through the curved cephalic arch; 3) The percent low WSS is significantly influenced by the pulsatility that is introduced in the cephalic vein by the fistula; and 4) The geometric and hemodynamic conditions in the cephalic arch are influenced by diabetes and time to fistula maturation (TM). In addition to our primary hypothesis involving the relationship between low WSS and NH and stenosis, other WSS-related quantities are also investigated to determine their role if any.

**Fig 1:**
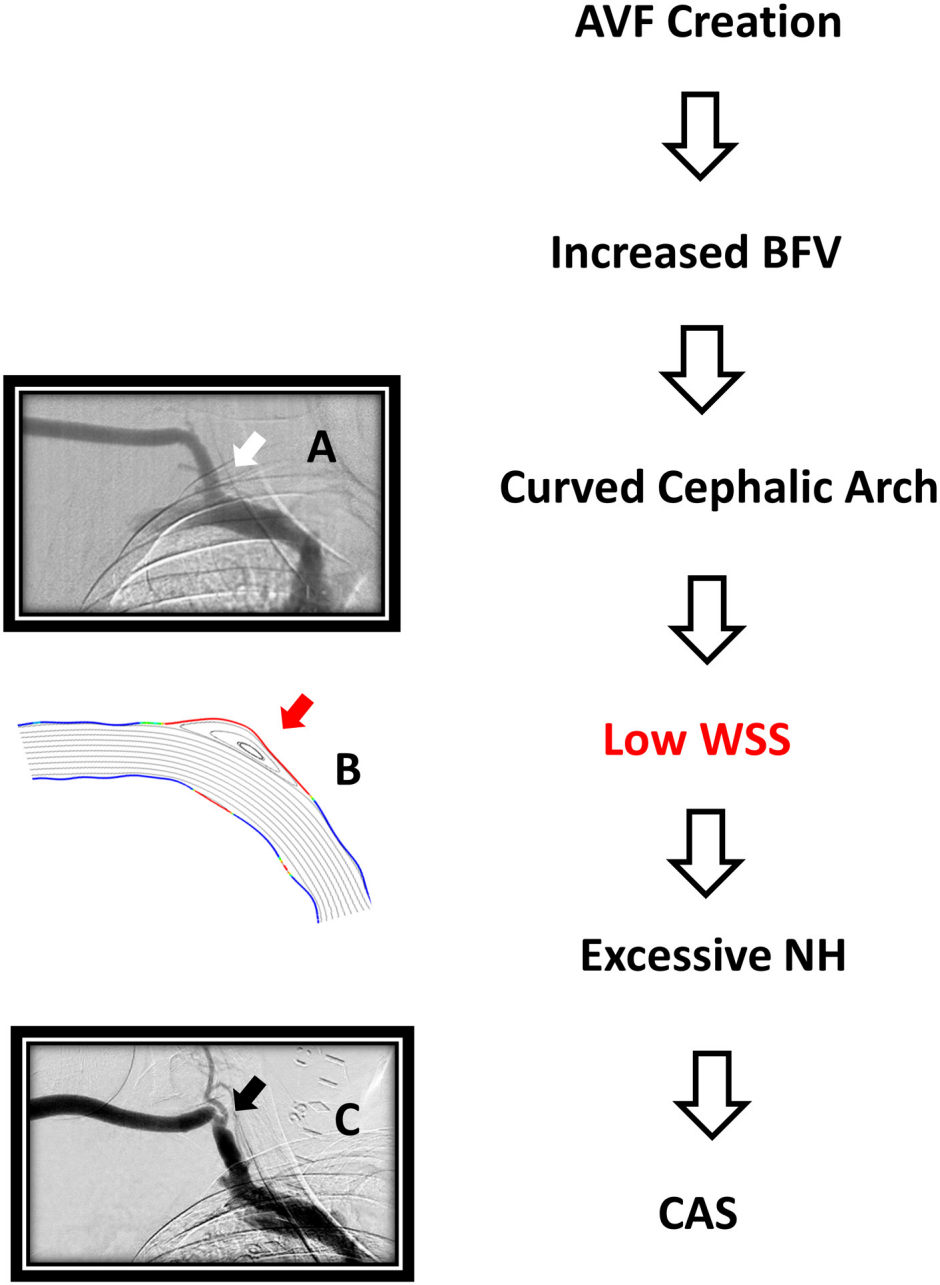
Schematic of hypothesis. Arteriovenous fistula (AVF) creation leads to high blood flow velocity (BFV) diverted into the curved cephalic arch (Panel A black arrow shows normal curved cephalic arch). Low wall shear stress (WSS) (bold red on upper and lower wall) causes recirculation areas represented in Panel B (red arrow). Low WSS will eventually cause excessive neointimal hyperplasia (NH) and resultant cephalic arch stenosis (CAS) (Panel B black arrow).

The overall aim of this work is to establish a relationship between BFV and low WSS in the cephalic arch of patients with BCF. In summary, the hypothesis is that high BFV at time of AVF creates high WSS in straight and curved segments of the cephalic vein. High WSS is needed for vein dilation and remodeling necessary for AVF maturation. Regions of low WSS develop in areas of the curved cephalic arch and predispose to endothelial cell injury with resultant biologic response of excessive NH and resultant venous stenosis (Fig 1). Venous stenosis develops in the cephalic arch, which then contributes to AVF failure. Computational modeling provides evidence that BFV predicts low WSS in the cephalic arch within months after fistula creation. Intervention to halt CAS may need to be directed at limitation of BFV.

## Materials and Methods

### Trial Design

This prospective observational trial was conducted at a single center university-affiliated medical center, from December 16, 2011 through January 22, 2014. The protocol was approved by the Institutional Review Board from the University of Chicago (Protocol number: 11–0269) on August 10, 2011. The study has maintained open enrollment with IRB approval since this time. The original trial study protocol that was approved prior to enrollment is available as supporting information (see S1 Appendix). The trial was conducted with good clinical practice and the Declaration of Helsinki. The trial was registered at ClinicalTrials.gov (NCT 01693263) August 8, 2012. The reason for the delay in registering the trial was that this protocol is an observational trial and did not fit the ICJME definition of a clinical trial and this policy. The trial was however registered to increase exposure making the trial open to all those who could benefit from it. The authors confirm that all ongoing and related trials for this intervention are registered. Informed consent was obtained in writing prior to any trial-related activities.

### Protocol

**Fig 2:**
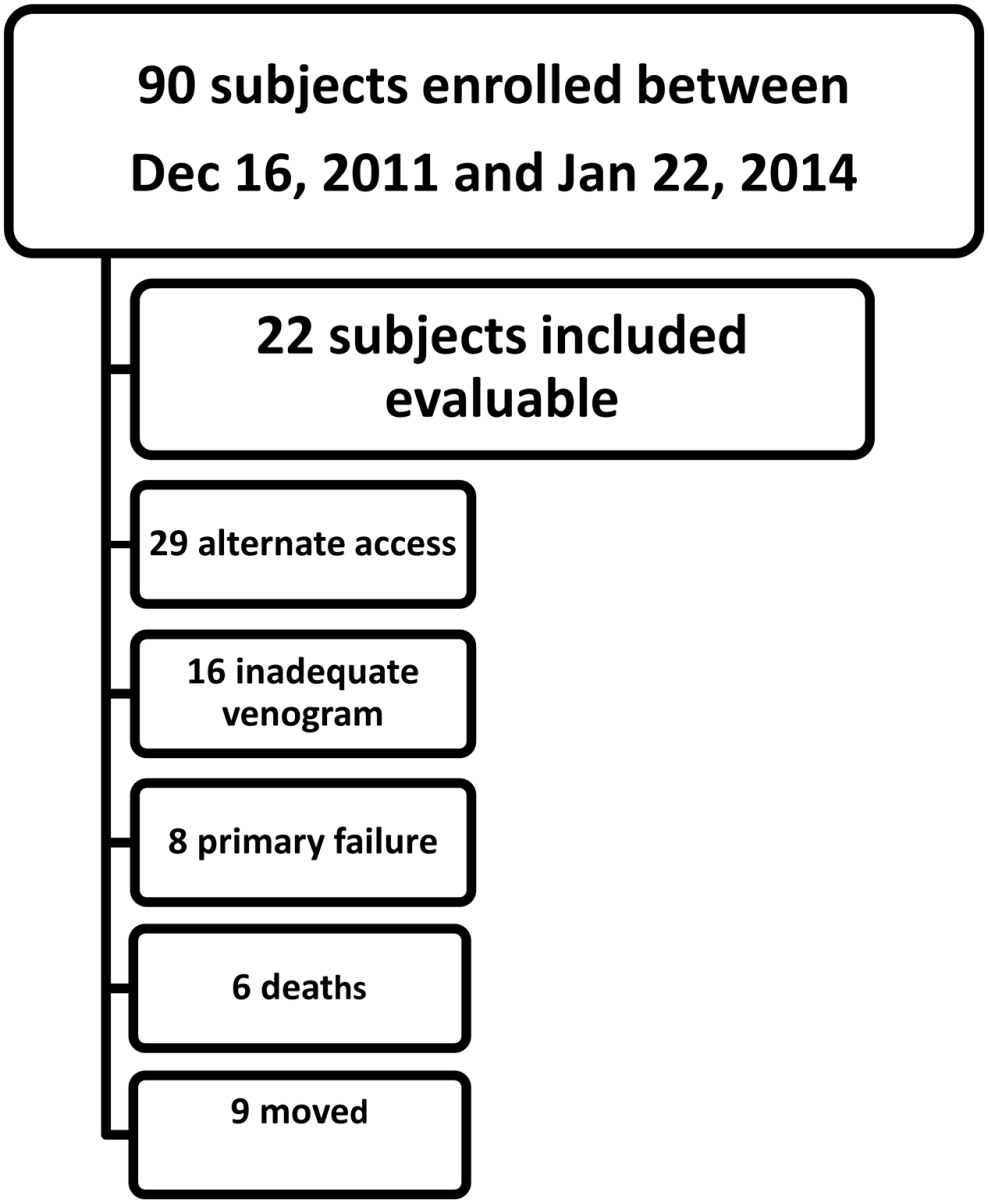
Study subject inclusion and discontinuation. Of 90 subjects enrolled, 22 had venograms at mapping suitable for measurements required in this study. 29 had alternate access placed, 16 had inadequate anatomy for geometric evaluation (such as a bifid arch), 8 had a primary failure prior to 3 month venogram, 6 deaths which were unrelated to the study and 9 subjects moved or had a change of modality.

This study represents a cohort of a larger population enrolled to determine the hemodynamic consequences of the post-fistula environment. Patients were consented to enroll in the study if they had advanced renal failure or were on hemodialysis in need of permanent access placement with plan for a BCF. During this study period, 90 subjects were enrolled, although only 22 of the subjects had baseline venograms performed prior to operative AVF creation (Fig 2). Although preoperative mapping venograms are often done as standard of care to identify suitable veins for AVF placement, the technique may result in suboptimal images for interpretation [17]. The subjects included had the following procedures performed at mapping and at time of maturation (8–32 weeks) with images allowing for measured parameters for geometric and computational modeling: a venogram, Doppler BFV measurement at the inflow of the cephalic arch, and blood sample for whole blood viscosity (WBV). Fistula maturation was defined as adequate arterialization of the venous segment and was determined clinically by evidence of a straight segment of palpable vein, a thrill at the arterial anastomosis continuing through the outflow vein, and a low pitched continuous bruit on auscultation. Clinically, all AVF studied at time of maturation were being used for hemodialysis with two-needle cannulation at a blood flow rate of at least 300 mL/min.

The patient had a fistula created as follows: the cephalic or the median cubital vein was dissected free using a combination of electrocaudary, blunt, and sharp dissection. The brachial artery was isolated in a similar fashion. The proximal end of the vein was controlled. The distal end of the vein was ligated, and the vein was divided. The artery was controlled both at the proximal and distal aspect. A longitudinal arteriotomy was made which was irrigated with heparinized saline, and an end vein to side artery anastomosis was completed. Vascular control was released. There was a Doppler signal only in the outflow vein and a palpable pulse in the artery distal to the anastomosis. The wound was irrigated and the incision was closed.

### Clinical Measurements

The venogram was performed with simultaneous BFV measurements via Doppler ultrasound by an Interventional Radiologist. For the mapping venogram, a peripheral IV was placed in the dorsum of the hands bilaterally. Contrast was injected via the peripheral IV, and sequential images from the wrist to the central vasculature were obtained. CO2 contrast was used if the patient was not on hemodialysis. A follow up venogram was performed when the fistula was determined to be clinically mature. The fistula was punctured using a micropuncture system near the arterial anastomosis with contrast injection towards the venous limb. The needle was exchanged for a 5 French dilator, and a digital subtraction venogram encompassing the outflow from puncture site to the right heart was performed. No significant difference in measured arch angle with position change of the arm was noted in a subset of 6 subjects (*p* > 0.05). TM was defined as the number of weeks between date of surgical placement and date of venogram taken when the patient met the criteria for fistula maturation.

Doppler spectral analysis was performed by an interventional radiologist at the time of each venogram to measure BFV in the cephalic vein. The peak systolic velocity at a 60-degree angle of insonation was measured in the straight portion of the cephalic vein between the anastomosis and the arch approximately 10 cm proximal to the cephalic arch. The velocities in this location were measured over several heart cycles and the average calculated.

WBV was measured from blood samples obtained from patients at baseline and TM. The blood was drawn during IV insertion on the day of the venogram and collected in 4.5 mL tubes containing 3.2% buffered sodium citrate. WBV was measured using a Brookfield programmable DV–II + cone and plate viscometer. The WBV was measured at various shear rates from 10 s^-1^ to 780 s^-1^ at 37°C. The shear rates were chosen between 10% and 100% of torque [18], and three measurements for each shear rate were determined.

### Measured Parameters

The venous diameter was measured in the straight portion of the vein, proximal to the arch, using a ruler placed in the field of study during the venogram. The arch angle was measured from the intersection of two straight lines, the first is a straight line drawn in the inflow segment and the second is a straight line drawn at the end of the arch at the junction with the axillary vein as previously described [14].

A novel parameter was derived to characterize the overall global curvature of the arch based on integrals of the local curvature of the cephalic vein walls. The two-dimensional venogram image was used to extract planar curves through image processing that represent the upper and lower vessel walls. These planar curves were provided explicitly as functions, i.e. *y* = *f*(*x*). The local curvature of a planar curve is defined as

$$k\left(x\right)\;=\;\frac{\left|y^{\prime \prime }\right|}{{\left(1+y\prime^{2}\right)}^{3/2}}$$

where *y*′ and *y*″ are the first and second derivatives of wall function *y* with respect to *x*, respectively. We integrated the local curvature over the total length of each vessel wall and then averaged the two results to obtain the Global Curvature parameter. Let *k*_1_(*x*) and *k*_2_(*x*) be the local curvature of the upper and lower walls, respectively. Thus,

$$\text{Global Curvature}=\frac{1}{2}\left[\int k_{1}\left(x\right)dx+\int k_{2}\left(x\right)dx\right]$$

A straight vessel has a Global Curvature of zero, and larger values indicate that the overall curvature of the vessel is more pronounced and/or the vessel is more tortuous. That is, the Global Curvature parameter accounts for both the global geometry of the arch, given by the arch angle, as well as the local behavior of the vein throughout the arch. It parameterizes more detailed information about the vessel geometry than the tortuosity parameter often used in arterial studies [7], which only parameterizes the centerline of the vessel. A second parameter was derived that represents the asymmetry between the upper and lower vein walls by taking the absolute value of the difference of the curvature integrals. Thus, a Global Asymmetry value of

$$\text{Global Asymmetry}=\frac{1}{2}\left[\int k_{1}\left(x\right)dx-\int k_{2}\left(x\right)dx\right]$$

zero means that the two vessel walls are perfectly symmetric, that is, parallel. Change in Global Curvature and Asymmetry was defined as the difference in the parameter from mapping to TM.

### Computational Model

A computational model of the hemodynamics through the cephalic vein and arch was created from the measured Doppler BFV, blood viscosity, and venogram image. This methodology has been used in previous research to predict various hemodynamic parameters such as velocity, pressure, and WSS throughout the vessel as shown in Boghosian et al. [19]. In the present investigation, two-dimensional simulations are carried out with and without pulsatility in order to determine its effect on the percent low WSS within the cephalic arch region. Recall that the normally non-pulsatile flow through the cephalic vein becomes pulsatile upon introduction of the arterial flow and pressure into the vein.

The mathematical model governing the hemodynamics in the cephalic vein and arch is the unsteady, incompressible continuity and Navier–Stokes equations, for which the governing equations in non-dimensional form are:

∇⋅v=0,

$$\frac{\partial v}{\partial t}+v\cdot \nabla v=-\nabla p+\frac{1}{Re}\nabla^{2}v,$$

where **v** is the velocity vector with components *u* and *v*, *p* is the pressure, and *Re* is the Reynolds number, which depends on the vessel size, inlet velocity, and blood viscosity. The flow of blood in the cephalic vein is taken as a homogeneous, Newtonian fluid, and the vessel walls are assumed to be rigid. Lee and Xu [20] considered the effect of vessel wall compliance on WSS and other flow features in a stenosed vessel. The vessel wall was considered to be composed of an isotropic material with uniform properties. The study found that WSS predictions were qualitatively the same between the rigid and compliant models, with the maximum difference in WSS being 7.1%. Non-Newtonian effects on the flow through the cephalic arch have been considered in a companion study by Mahmoudzadeh Akherat [21]. It is found that the patient-specific geometry has a much greater influence on the locations of low WSS regions, and the non-Newtonian effects only have a minimal influence on the extent of the recirculation regions.

Boundary conditions need to be imposed at the inlet, outlet, and walls of the vessel. At the inlet we impose a parabolic profile based on the Doppler velocity measurements in the form

$$u\left(y\right)=\frac{3}{2}\left(1-y^{2}\right),\;v=0.$$

The cephalic vein is relatively straight upstream of the computational domain; therefore, a parabolic profile is a good approximation. In addition, the hemodynamic results within the cephalic arch are not sensitive to the inlet velocity profile due to the long, relatively straight portion of the vein that exists between the inlet to the domain and the start of the arch. On the walls we enforce the no-slip and impermeable conditions, such that *u = v = 0*. At the outlet location, the pressure is not known a priori. As a result, a standard pressure outflow boundary condition is used. For the initial condition, the flow is assumed to start from rest, and the unsteady solution is evolved in time until either a steady solution is obtained or a clear unsteady flow pattern develops. The flows at mapping and maturation are steady as the Reynolds numbers are insufficient to produce unsteady and/or transitional flows in our cohort of 22 patients, even considering the irregular wall geometries.

Our intent is to show that increasing the flow rate through the cephalic arch results in an increase in the regions of low WSS. Although a three-dimensional model would provide more detail of the hemodynamics within the cephalic arch, the two-dimensional model used here is sufficient to indicate the regions within the arch that are subject to low WSS using clinically available imaging technology. Note that a three-dimensional model would require intravascular ultrasound (IVUS) or MRA imaging, which are not widely clinically available. A detailed comparison of the two- and three-dimensional models will be the subject of a subsequent paper, which will show that the two-dimensional model is conservative with regard to the low WSS relative to the three-dimensional model.

Grid resolution convergence studies have been performed to ensure that the results are accurate and grid independent. For the simulations below a Reynolds number of approximately 700, the average number of degrees of freedom is close to 150,000. For Reynolds numbers above 700, there are approximately 300,000 degrees of freedom. These values result in reasonable grid-independence with differences between grids of only a few percent.

From our computational model, the WSS throughout the cephalic vein is determined. Of primary interest in this investigation are the locations where the WSS is low enough to produce NH. The fraction of the cephalic arch wall affected by low WSS is reported along with the locations where this is the case. Specifically, the entire computational domain consists of an approximately 10 cm portion of the cephalic vein that starts downstream of the anastomosis in a relatively straight segment of the cephalic vein. The model extends to the downstream end (with respect to flow direction) of the cephalic arch, which ends at its juncture with the axillary vein. As the length of the domain varies from patient to patient, the percent low and high WSS is reported only in the arch portion, i.e. where the straight portion curves to join with the axillary vein. Critically high WSS can cause denudation of the endothelial cells; therefore, the fraction of the arch so affected is tabulated as well. In addition to low and high WSS, the computational data also allows for determination of the maximum spatial WSS gradient throughout the cephalic arch. This is the spatial derivative (rate of change) of the WSS with distance along the vein wall.

### Data Analysis

Continuous variables were summarized using means and standard deviations. Counts and percentages were used to summarize binary variables. Scatterplots and correlation coefficients were used to examine pairwise relationships among study variables. Parameter measurements at baseline and time of maturation were compared via Wilcoxon signed rank tests. Relationships among arch angle, flow velocity, percent low WSS, and maximum spatial WSS gradient were examined using linear regression. Linear regression was also used to investigate whether diabetic status, gender, age (≤ or >58.5 years), BMI (≤ or >30), or history of CAD or PVD moderated the relationships between TM and changes in measured parameters from baseline. The Benjamini-Hochberg method of *p*-value adjustment was used to control the false discovery rate at 5%.

Within-patient log-transformed wall shear stress values were summarized separately for the lower and upper wall using descriptive statistics and boxplots. Each patient’s lower and upper WSS values were compared using a non-parametric two-sample Wilcoxon rank sum test, with p-values adjusted using the Benjamini-Hochberg method to control the false discovery rate for the 12 statistical tests. Overall, lower and upper wall values were compared across patients using a repeated measures ANOVA applied to the raw and rank-transformed WSS data. All statistical analyses were performed in R version 3.0.2 (http://www.r-project.org/).

## Results

### Study Population

Twenty-two subjects completed sufficient observations to be included in this investigation (Table 1). Patient demographics and past history were obtained from electronic medical records. A patient was considered to have CAD if they had a prior history of myocardial infarction or cardiac catheterization showing significant coronary disease. A patient was considered to have PVD if they had a history of amputation from ischemic disease or a vascular study showing ankle-brachial index less than 0.9.

**Table 1:** Subject Characteristics.

Patient ID	Age (yr)	Sex	Ethnic	Weight (kg)	Height (cm)	BMI	TM (wks)	Diabetic	HTN	CAD	PVD
1	59	M	Cau	90.1	180.0	27.7	9	-	+	+	-
2	45	M	AA	94.4	170.2	32.6	8	+	+	-	+
3	61	F	AA	57.7	172.7	19.4	15	-	+	+	-
4	65	F	AA	74.9	152.4	32.3	30	-	+	-	+
5	66	F	AA	86.3	160.5	33.5	9	+	+	+	-
6	91	M	AA	72.6	182.9	21.7	19	+	+	+	+
7	71	M	Cau	104.2	182.9	31.2	30	+	-	+	+
8	66	F	AA	90.6	170.2	31.3	17	+	+	+	-
9	55	F	AA	134.1	161.0	51.7	16	+	+	-	+
10	76	M	Cau	83.5	167.6	29.7	13	+	+	+	+
11	63	F	AA	50.9	156.2	20.9	13	-	+	-	-
12	45	M	AA	85.1	165.1	31.2	28	-	+	+	-
13	70	F	AA	76.9	154.5	32.2	9	+	+	-	-
14	31	F	AA	54	140.0	27.5	16	+	+	-	-
15	61	F	Cau	62.7	142.2	31.0	14	+	+	-	-
16	38	M	AA	71.2	172.7	23.9	13	-	+	-	-
17	43	M	AA	104.9	172.7	35.2	21	-	-	-	-
18	61	F	AA	73.5	156.2	30.1	20	+	+	+	-
19	34	F	AA	103.5	160.0	40.4	15	+	+	-	-
20	47	F	AA	195	170.2	67.3	14	+	+	-	-
21	58	M	AA	99.3	175.3	32.3	17	+	+	-	-
22	24	F	Cau	43.6	149.9	19.4	15	-	-	-	-
	55.9 ± 16.1	9 (41%)^1^	5 (22%)^2^	86.8 ± 32.1	164.4 ± 12.1	31.9 ± 10.7	16.4 ± 6.3	14 (64%)	19 (86%)	9 (41%)	6(27%)

Values are means ± SD or count (%) as appropriate. yr = years; F = female; M = male; Cau = Caucasian; AA = African American; kg = kilograms; cm = centimeters; BMI = Body Mass Index; TM = time to fistula maturation; wks = weeks; HTN = hypertension; CAD = coronary artery disease; PVD = peripheral vascular disease;

^1^ denotes male;

^2^denotes Caucasian

We chose all subjects with the need for primary BCF who were either on hemodialysis or had an access placed as it was anticipated that they would need dialysis within the next 6 months. Eighteen of the subjects were receiving dialysis through an alternative access at the time of fistula placement. All ESRD patients who were receiving hemodialysis three times a week achieved average single pool Kt/V of at least 1.2 over three months.

### Wall Shear Stress

**Fig 3:**
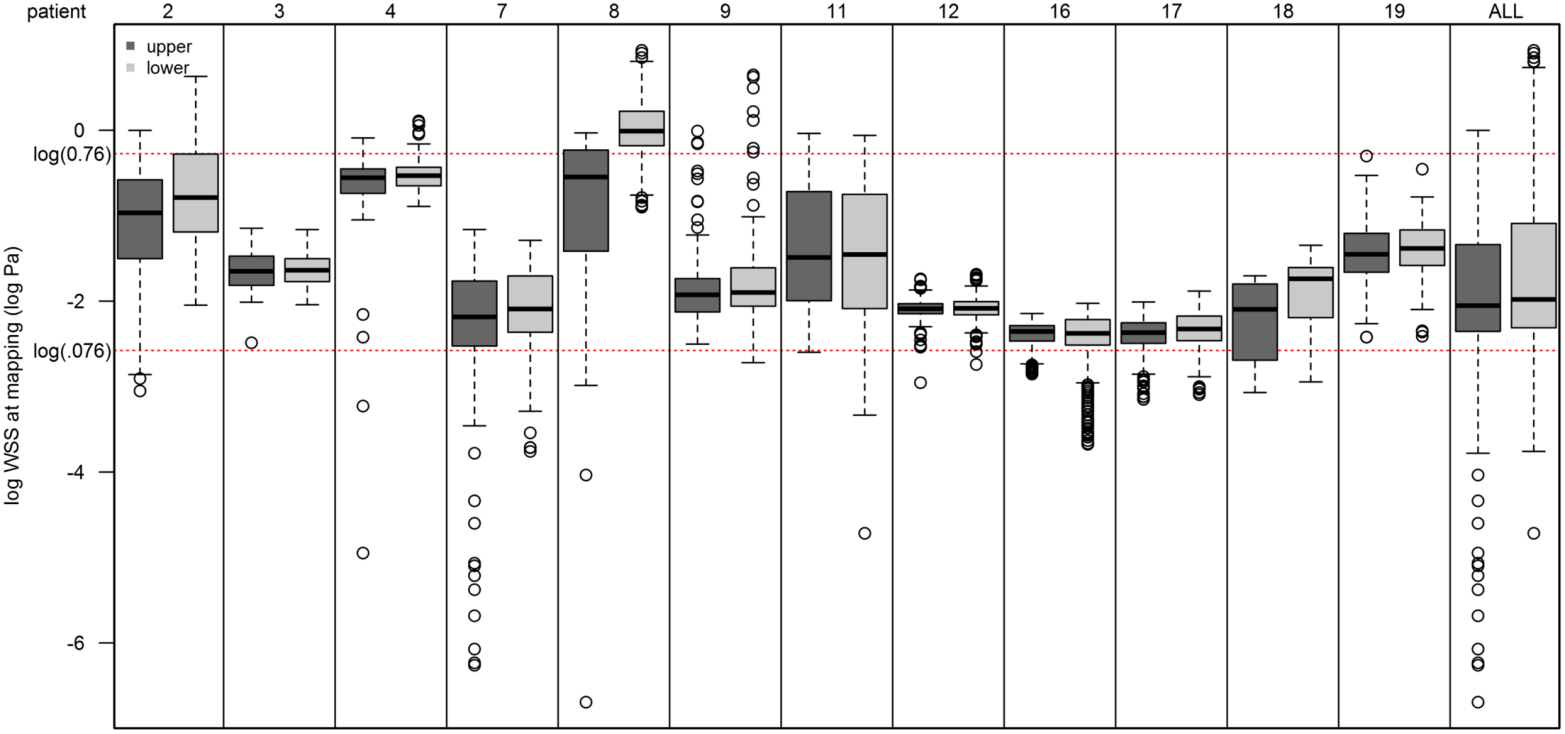
Wall shear stress at mapping in 12 subjects. WSS (log *Pa*) at mapping for 12 subjects. Within-patient log-transformed wall shear stress values in the upper (dark grey) and lower wall (light grey). Red reference lines show the normal range on the original scale log scale [log(0.076)-log(0.76)].

Normal WSS for veins has been referenced to be 0.076–0.76 *Pa* for medium to large veins [22,23]. To validate this reference range in the current study, 12 patient-specific computations were performed at pre-access (mapping) with WSS determined for the upper and lower vein walls. Values within the range provided in the literature were evident in 80 and 86% of the lower and upper wall measurements, respectively. The average WSS for the upper wall was 0.23 *Pa* and 0.3 *Pa* for the lower wall, which falls well within the reference range of 0.076–0.76 *Pa* (Fig 3). Upper wall WSS values tend to be about 0.0758 units lower than the upper wall, which was significant (*p* < 0.001). Given these findings, a low WSS was defined as less than 0.076 *Pa*. Critically high WSS is defined as being above 40 *Pa*, which is the threshold above which denudation of the endothelial cells begins to occur [24].

**Fig 4:**
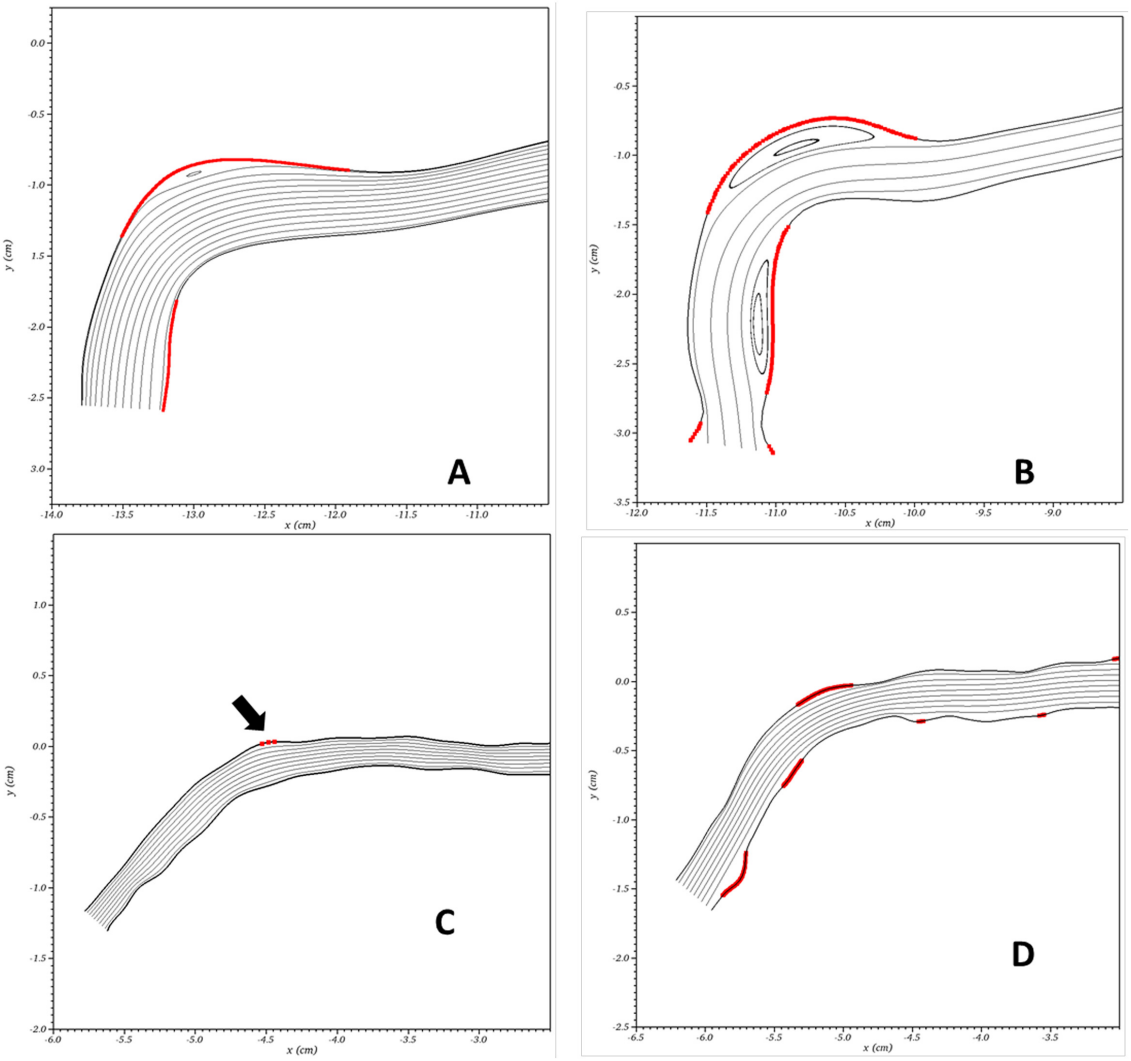
Computational flow plot of cephalic arch. Terminal cephalic arch with inflow from right to left. Critical wall shear regions (< 0.076 Pa) are denoted with bold, red lines and are superimposed on streamlines. Computational flow plot for subject 7 at baseline (Panel A) and 30 weeks (Panel B). Computational flow plot for subject 2 at baseline (Panel C) and 8 weeks (Panel D). Black arrow on Panel D shows tiny area of low wall shear stress.

The specific areas having low WSS within the arch are shown in Fig 4 for two patients. These two patients were chosen as they represent the extremes of maturation: patient 7 (Tables 1 and 2) achieved maturation by 30 weeks, whereas patient 2 (Tables 1 and 2) achieved maturation at 8 weeks. Panels A and C show the geometries and hemodynamics at baseline for patients 7 and 2 (Tables 1 and 2), respectively, while Panels B and D show the same results at TM. In these figures, bold red lines indicate regions where the WSS is below the lower limit of the normal physiologic range for medium to large veins (< 0.076 *Pa*) [22,23].

**Table 2:** Patient Measured Parameters.

	% low WSS	Pulsatile % low WSS	Velocity (cm/sec)	% Critically high WSS	Spatial WSS	WBV (cP)	Global Curvature		Global Asymmetry		Arch Angle (°)		Venous Diameter (cm)	
Patient	TM	TM	TM	TM	TM	TM	Baseline	TM	Baseline	TM	Baseline	TM	Baseline	TM
1	12.18	8.40	54.33	0.25	2895.7	3.28	1.60	1.79	0.17	0.31	119	120	0.36	0.45
2	19.39	9.13	94.17	0.34	26619	3.44	1.52	2.54	0.35	1.01	134	119	0.21	0.40
3	1.74	0.53	14.27	0	156.9	2.99	1.06	1.74	0.21	0.53	148	139	0.35	0.40
4	10.95	3.52	49.73	0	2706.4	2.91	1.49	1.79	0.25	0.25	116	107	0.10	0.39
5	17.88	16.15	38.07	0	2220.1	3.36	1.82	1.33	0.53	0.34	133	116	0.37	0.33
6	14.67	12.10	44.33	0	171.5	2.97	2.20	2.02	1.43	0.70	134	105	0.14	0.50
7	39.73	35.3	17.93	0	2118.9	3.16	2.48	3.57	0.17	1.56	114	103	0.35	0.44
8	13.19	11.02	36.13	0	203.1	3.75	2.65	2.36	1.09	1.48	137	129	0.10	0.30
9	38.80	19.94	83.67	0.63	5006.6	3.35	1.79	1.55	0.20	0.24	130	118	0.45	0.64
10	2.64	4.73	6.43	0	811.5	3.47	2.52	2.03	1.33	0.02	142	93	0.28	0.40
11	20.56	15.30	79.43	0	4025.2	3.51	2.55	2.12	1.28	0.14	156	126	0.36	0.45
12	38.10	36.10	83.20	0	8858.5	2.48	1.54	2.46	0.12	1.55	116	107	0.30	0.30
13	12.60	12.00	125.00	0.05	6095.4	3.11	3.05	1.64	1.18	0.03	93	120	0.27	0.45
14	19.60	12.62	81.40	0.5	7881.1	3.12	1.56	1.85	0.55	0.33	135	132	0.11	0.50
15	30.71	24.70	16.33	0	7620.7	2.67	4.09	3.17	0.80	0.43	112	111	0.25	0.50
16	28.00	34.50	83.30	0	12147.5	3.48	1.28	1.5	0.56	0.38	130	149	0.42	0.65
17	66.72	65.80	99.77	0.24	9349.8	2.67	3.17	3.32	1.80	0.12	125	128	0.40	1.00
18	5.53	4.12	33.74	0	451.7	3.09	1.52	1.83	0.50	0.47	100	115	0.35	0.43
19	10.39	7.10	54.33	0	1132,7	2.96	3.54	3.58	2.49	1.85	126	120	0.22	0.48
20	56.54	51.13	143.80	0	8912.1	2.89	1.53	1.68	0.03	0.17	96	144	0.70	0.80
21	9.01	13.85	30.47	0	614.8	3.25	1.12	1.91	0.00	0.30	121	134	0.35	0.45
22	41.28	33.64	76.40	0	9798.8	2.88	2.49	1.94	0.63	0.36	105	124	0.16	0.65
	22.87±17.1	19.67±16.8	61.2±36.8	0.09 ± 0.18	5445.36±6061.1	3.13±0.32	2.12±0.8	2.17±0.7	0.71±0.7	0.57±0.5	124±16.4	121±13.8	0.29±0.1	0.5±0.2*

Values are means ± SD. % low WSS = percent low wall shear stress; WBV = whole blood viscosity; TM = time to maturation

*p < 0.05 comparing baseline to TM

As with time to maturation, the computational results (Fig 4) represent two extremes hemodynamically. Patient 7 has an acute arch angle at baseline (Panel A) resulting in two regions of low WSS with a large recirculation region visible in the streamlines near the upper elbow of the arch. In the same subject, 30 weeks of maturation (Panel B) has caused changes in wall geometry that have led to more prominent recirculation regions with associated regions of low WSS. This patient’s cephalic arch already had a large diameter and acute angle at baseline (Table 2). In patient 2, the diameter of the cephalic vein nearly doubles from time of placement (Panel C) to TM (Panel D), and the arch angle is significantly less acute at baseline (Panel C) than at TM (Panel D). The more gradual arch at placement exhibits no recirculation regions and only one tiny region of low WSS indicated by the arrow (Fig 4, Panel C). After 8 weeks of maturation (Fig 4, Panel D), this same patient shows a massive increase in the fraction of vessel wall having low WSS (Panel D).

As there is no guidance from previous investigations to provide values of spatial WSS gradient that may be physiologically or clinically relevant, we simply report the maximum values for each patient within the arch in Table 2. Although these maximum values vary linearly with BFV (r = 0.75, p < 0.001), it is unknown if the locations where the maximums occur correspond to areas in the arch where NH and stenosis will eventually occur.

### Pulsatility

**Fig 5:**
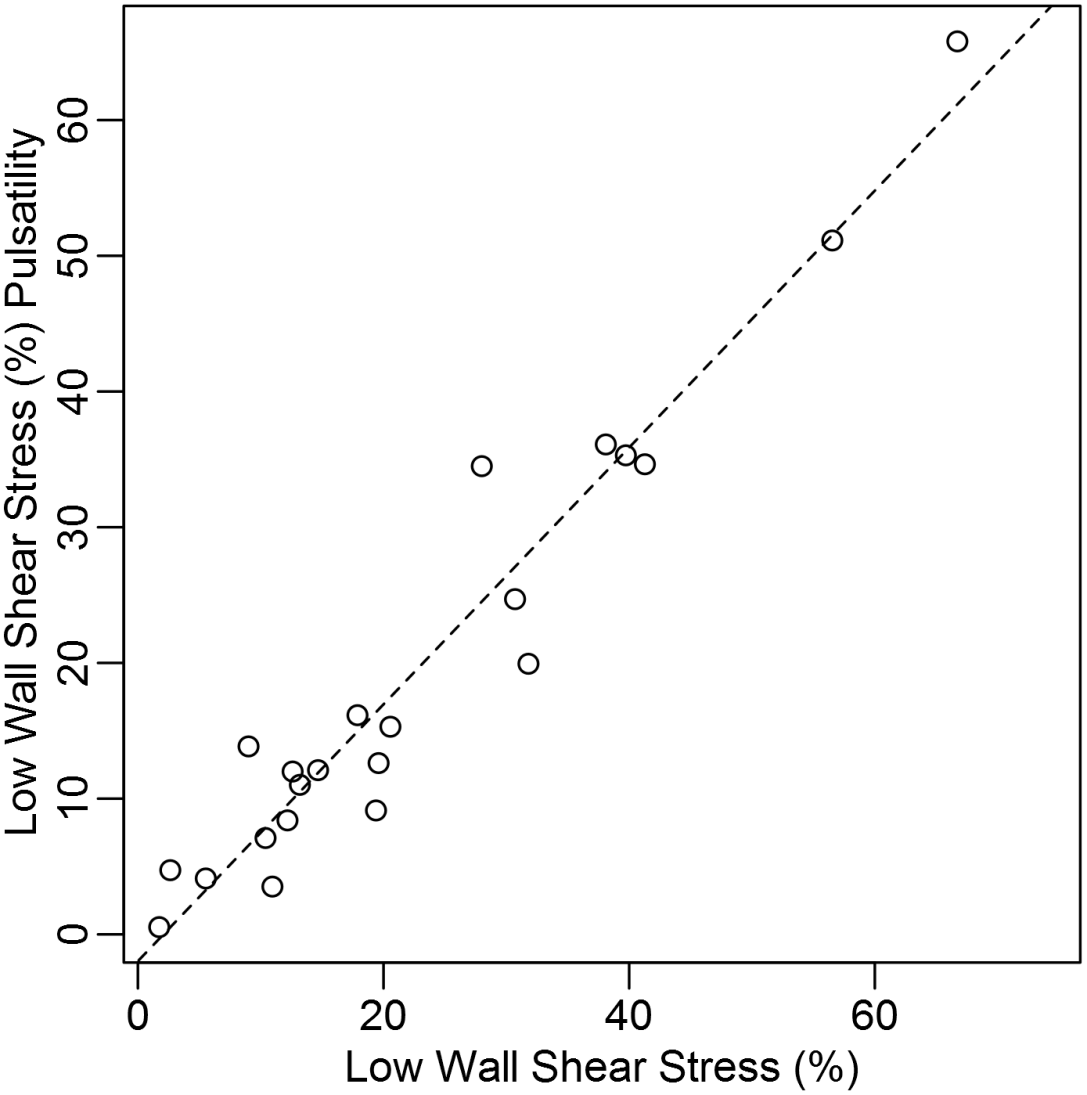
Scatterplot depicting relationship of percent low WSS and cycle average percent low WSS. Percent low WSS in arch is shown on the *x*-axis with cycle average percent low WSS shown on the *y*-axis. The percent low WSS are strongly correlated with cycle average percent low WSS (*p* < 0.001).

Our standard protocol is to average the maximum velocity during three cardiac cycles from the Doppler velocity measurements to use as the steady inlet for the hemodynamic simulations, thereby modeling the worst-case scenario. However, the Womersley number for the pulsatile flow through the cephalic arch after maturation is typically between two and four, which are inconclusive with regard to the influence of the pulsatile frequency on the hemodynamics [25]. Therefore, in order to determine the influence of pulsatility on the hemodynamics, in particular the regions of low WSS, all cases considered in this investigation were recalculated using actual patient-specific cardiac waveforms taken from the time-dependent Doppler measurements and used as the unsteady inlet velocity for each patient. When the percent low WSS in the cephalic arch with steady inlet was compared to percent low WSS in the arch with pulsatility, there was a significant difference (*p* = 0.00043); however, the two measurements were shown to have a positive correlation (*r* = 0.92, *p* < 0.001) (Fig 5). The average difference in percent low WSS in the arch between pulsatile and steady inlet conditions was 3.2%.

### Hemodynamic Conditions

**Fig 6:**
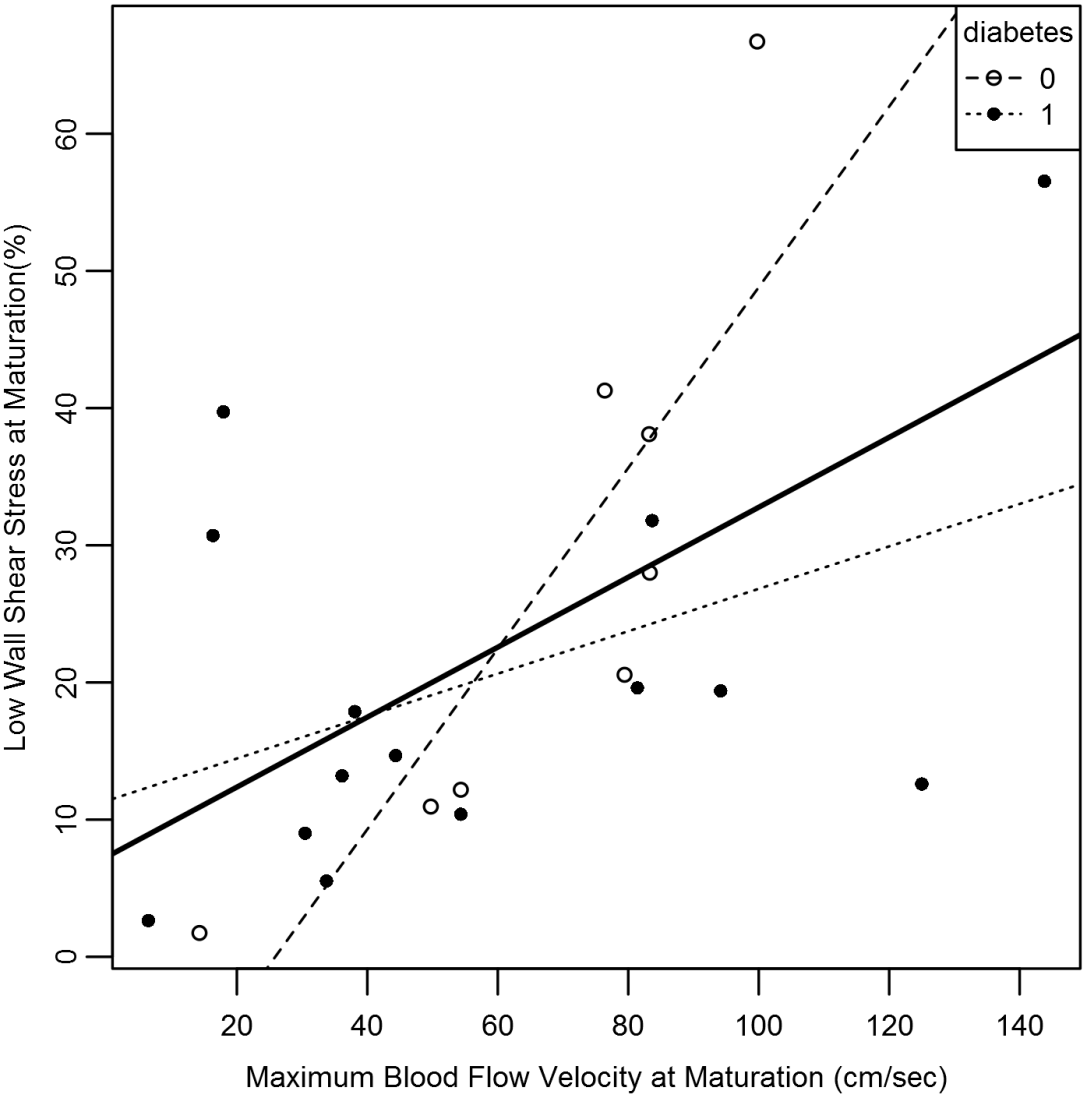
Scatterplot depicting the relationship of blood flow velocity and WSS at maturation. Maximum blood flow velocity (cm/sec) is shown on the *x*-axis and percent low wall shear stress is shown on the *y*-axis. Blood flow velocity is correlated with changes in low wall shear stress (solid line) (*p* < 0.05). The patients with diabetes are represented by closed circles, the patients without diabetes by open circles with significant correlation in non-diabetics (*p* < 0.05).

The percent low WSS was linearly related to the BFV at TM. (Fig 6; slope 0.66; *r* = 0.55; *p* = 0.0084). The percent low WSS (*p* = 0.41) and BFV (*p* = 0.5) did not differ between diabetics and non-diabetics at maturation. However, for every unit increase in BFV, the expected increase in percent low WSS was greater for non-diabetics (slope 0.66, *r* = 0.85, *p* = 0.008) than for diabetics (slope 0.15, *r* = 0.44, *p* = 0.12) (Fig 6), but not for gender, age, BMI, or history of CAD or PVD (*p* > 0.05, all comparisons). The relationship between arch angle and blood flow velocity was not significant for the overall population studied (*p* = 0.26) but showed a difference when comparing diabetes and non-diabetics (*p* = 0.05). The relationship between venous diameter and BFV was significant when the overall population was studied (*p* = 0.02) but did not show a difference when comparing diabetics and non-diabetics (*p* = 0.16).

### Venous Shape Parameters

Venous diameter increased in all subjects except two, with a mean difference of 0.21 ± 0.16 from baseline to TM (*p* < 0.001) (Table 2). Although individual parameters show that the arch angle decreased in 13 subjects from baseline to TM, the mean difference of –2.86 ± 6.44 was insignificant showing no correlation with TM (*p* = 0.53).

**Fig 7:**
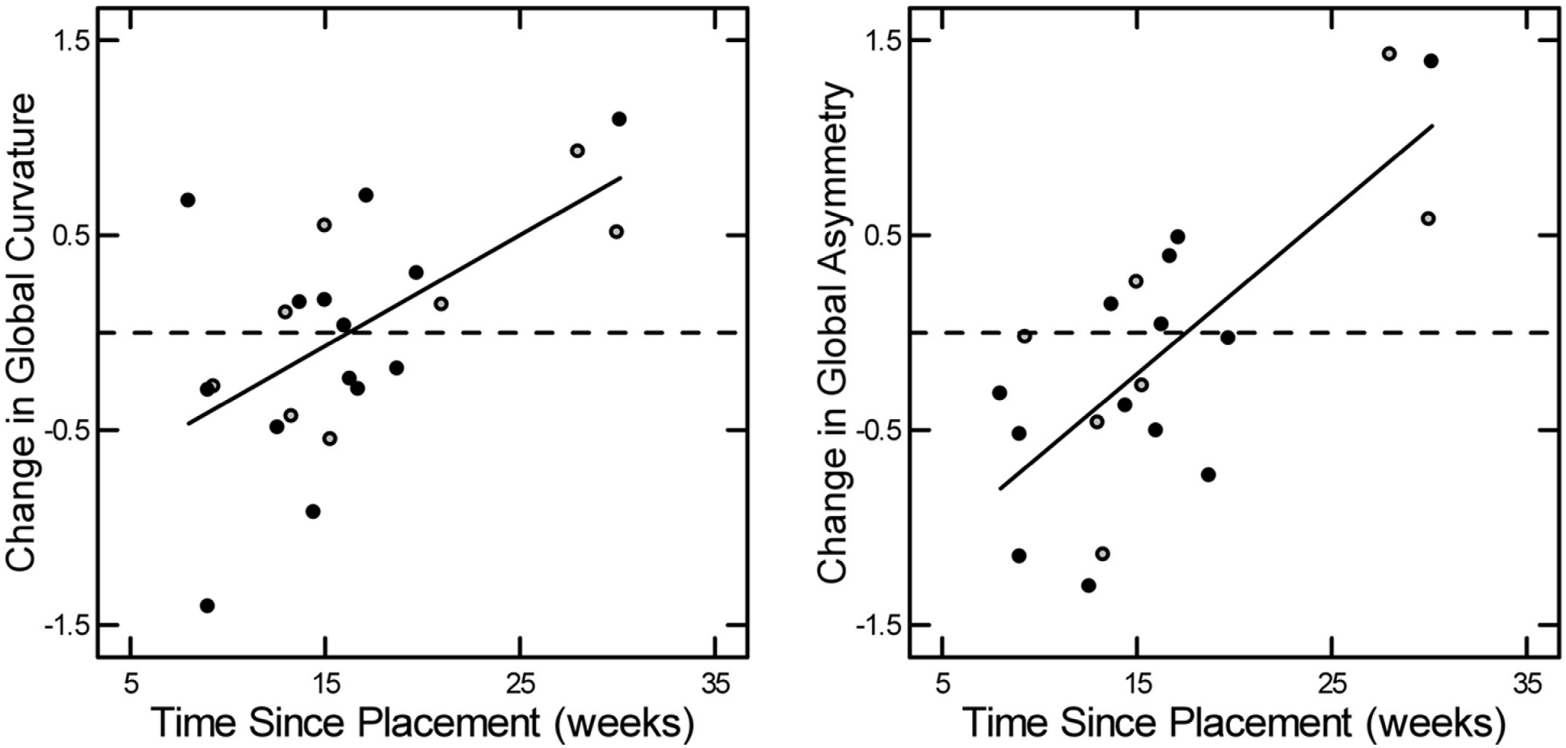
Scatterplots depicting the relationship between weeks since access placement and Global Curvature or Global Asymmetry. Time since access placement (weeks) is shown on the *x*-axis and change in Global Curvature and Global Asymmetry are shown on the *y*-axis. Changes in Global Curvature (left panel) and Global Asymmetry (right panel) increase with time since the fistula was placed (*p* < 0.05). The relationship did not differ significantly in patients with diabetes (closed circles) compared to those without diabetes (open circles) (*p* < 0.05).

A more complete description that incorporates overall arch angle and local geometry is provided by the Global Curvature (see METHODS). Although the mean Global Curvature for the entire population remained essentially unchanged from baseline to TM (*p* > 0.05) (Table 2), the change fell for short TM (Fig 7, left panel) and rose for longer TM (*r* = 0.52; *p* = 0.01). The regression line crosses zero at TM of 15–16 weeks.

The change in Global Asymmetry (see METHODS), our novel measure of variability in curvature between the upper and lower walls of the cephalic arch, also changed between baseline and TM (*r* = 0.47; *p* = 0.03) (Fig 7, right panel). For short TM, Global Asymmetry fell from baseline, and for longer TM rose above baseline. As for change in Global Curvature, the regression line crossed zero at TM of 16–17 weeks. Neither the cross products of diabetic status or gender with time since placement were significant (*p* > 0.05).

## Discussion

The results of this study clearly uphold our primary hypothesis that hemodynamic changes resulting from creation of the AVF lead to regions of low WSS. Our TM was chosen as the time an access was fully sufficient to support dialysis needs. At this crucial time, we easily demonstrated regions of low WSS in almost all of our patients. Our second hypothesis was that low WSS, i.e. less than 0.076 Pa, is caused by increased BFV through the curved cephalic arch. We showed that the regression of low WSS fractional coverage of the cephalic arch rises with increase of flow velocity. Had we failed to find this significant regression, the specific hypothesis relating increased BFV to regions of low WSS in the cephalic arch would have been disproved.

The normally non-pulsatile flow in the cephalic vein becomes pulsatile when the AVF is created. Our third hypothesis was that this pulsatility substantially influences the percent low WSS in the cephalic arch. This hypothesis was found to be false as the percent low WSS is primarily determined by the local geometric features that characterize the vein walls and only secondarily by the variations in BFV throughout the cardiac cycle. This result is somewhat surprising given the often large variations in blood flow throughout the cardiac cycle; however, it must be emphasized that the percent low WSS is the primary hemodynamic parameter of interest in this application.

Our fourth hypothesis is not crucial to our primary work, but it arose from clinical thinking: diabetes, being a cause of massive vessel disease, should affect CAS and its mechanisms for production. In fact, we found this to be true. As increased BFV causes low WSS, this may explain why clinically evident CAS is attenuated in diabetic patients with ESRD. Follow-up over time of the cohort studied will determine if this remains so.

In addition to the primary findings, geometric changes are found to occur during the maturation process. The venous diameter increased with increased blood flow at time of maturation, while the arch angle was shown to increase with blood flow velocity at time of maturation only in diabetics. The upper and lower walls of the cephalic arch show differences in hemodynamics. The upper wall of the cephalic arch had WSS that was less than that of the lower wall. The Global Curvature parameter captures the global change in angle and local tortuosity of the vessel. The companion Global Asymmetry parameter indicates if the upper and lower walls of the arch geometries are similar to each other. Smaller values for both Global Curvature and Asymmetry parameters are ideal, i.e. the vein and arch wall geometries are smooth and parallel. As shown in Table 2, there is no significant change in Global Curvature or Asymmetry from baseline to TM. However, there is a marked increase in the *change* in both parameters with TM. For short TM, the shape parameter values decrease from baseline; however, for a TM longer than 15 weeks, the maturation process introduces adverse geometric effects, i.e. new curvature and asymmetry. As a result, this critical time threshold of about 15 weeks may be clinically significant. These two novel parameters have been developed as part of this research and are being applied to cardiovascular systems for the first time. Follow up as to the differences in upper and lower wall geometries and hemodynamics is needed.

There are other factors that contribute to adequate maturation of the vein that are not accounted for in this study but will be the focus of future investigation. These include: underlying vein histology, adequate generation of nitric oxide, oxidative stress induced by dialysis itself and direct pressure to the endothelium [26, 27, 28]. BFV has been shown to be the major determinate in successful dilation of an AVF with pressure playing only a minor role [29]. The ability of the vein to remodel [30, 31] achieving intraluminal dilation is important to adequate fistula function. Our study does not address the intraluminal diameter, but future investigation with IVUS will allow this [32]. Additional limitation in the computational model includes its dependence on two-dimensional venograms. Two-dimensional computational analysis is unable to show such parameters as helicity, but it does enable a large number of subjects to be included and compared over time.

Given the clinical sequela of access failure, it is important to prevent the development of CAS. The novel finding in this study is that geometric and hemodynamic changes begin early after fistula creation due to the dramatically increased BFV through the curved cephalic arch. Although necessary to promote maturation, this increased BFV is beyond that required for successful access function and is damaging the veins in such a way as to reduce the long-term patency of the BCF. Once hemodialysis is started, there is even more flow generated through the arch, which may worsen the low WSS. The critical next step is to validate the clinical outcome of CAS in these patients and determine topographical coordination to see if the areas of low WSS develop into areas of CAS over time. If so, intervention to obviate CAS will need to be done early, possibly at the time of AVF creation or at a time before TM. An understanding of the molecular basis of signaling pathways induced by pressure and flow on the endothelium of veins after creation of an AVF are also needed to prevent the pathway of NH and ultimate CAS [33,34].

## Supporting Information

S1 Appendix Study Protocol for "A Clinical and Computational Study to Improve Brachiocephalic Fistula Outcomes". (PDF)
