# Supplementary material for: Increased Inlet Blood Flow Velocity Predicts Low Wall Shear Stress in the Cephalic Arch of Patients with Brachiocephalic Fistula Access
Source: PLoS One. 2016 Apr 13;11(4):e0152873. doi: 10.1371/journal.pone.0152873 (PMC4830603; doi:10.1371/journal.pone.0152873)
Supplement: S1 Appendix — (PDF) [file pone.0152873.s001.pdf]

A Clinical and Computational Study to Improve Brachiocephalic Fistula Outcomes

PI:Mary Hammes, D.O.

Sub-Investigators: IIT
Kevin Cassel cassel@iit.edu
Michael Boghosian boghmic@iit.edu
Promila Dhar dhar@iit.edu
Sandra Menezes smenezes@iit.edu

Sub-Investigators: U of C
Hae Kyung Im BSD Health Studies haky@uchicago.edu
Yolanda Becker ybecker@surgery.bsd.uchicago.edu
Brain Funaki bfunaki@uchicago.edu
Neeraj Jolly jolly@medicine.bsd.uchicago.edu
Fred Coe fcoe@uchicago.edu
Diane Fodor dfodor@surgery.bsd.uchicago.edu
Anthony Chang anthony.chang@uchospitals.edu

Background:

The outcome of patients with end stage renal disease (ESRD) on hemodialysis depends on a functioning vascular access. Although a variety of techniques have been developed for providing hemodialysis access, there have been no major advances for the past three decades. This contributes to the fact that hemodialysis access dysfunction is one of the most important causes of mortality in the hemodialysis population. In addition, the expense of providing ESRD care in the US is a significant portion of the Medicare budget, totaling $23.9 billion in 2007, of which a significant portion is spent on placement and maintenance of vascular access [1]. Not only does fistula access provide the best outcomes, it also can be placed and managed with the least expense as compared to catheters and grafts. Therefore, regional and national indicators promote the placement of arteriovenous fistulae (AVF). For example, the national vascular access initiative ``Fistula First" was developed to promote fistulae with an initial goal of placing 40% of prevalent patients with fistula access. This goal was achieved in 2005, and a goal of 66% was set for 2009. Nationwide, however, there are only 54.4% of prevalent hemodialysis patients with fistula access as of November 2009, and the number of fistula access placements fell for the first time in 2007 [1]. It is imperative to determine how fistulae can be improved, both in terms of outcomes and cost. Diabetics represent approximately 40% of those on dialysis, and it is projected that the number of people in the US diagnosed with diabetes will nearly double in the next 25 years, and the annual diabetes-related spending is expected to triple in the same time frame [2].

Native AVF have the least number of complications and are recommended for vascular access [1,3,4]. The AVF with the best outcomes is the radiocephalic fistula (RCF); however, this access often fails to mature in elderly patients with underlying vascular disease, particularly in diabetics [5,6]. The second recommended access is the brachiocephalic fistula (BCF), which has a mean duration of function of only 3.6 years in contrast to RCF, which often exceed 5 years [6].

The proposed investigation will address the primary mode of failure for dialysis patients with BCF, thereby further improving its viability relative to other access options.

Cephalic arch stenosis (CAS) commonly occurs in patients with BCF and contributes to loss of vascular access [7]. The leading cause of failure for BCF is due to stenosis in the cephalic arch, which is the final bend in the cephalic vein prior to entry into the axillary vein. In a retrospective study in our own facilities, CAS was found to occur in 77% of patients with BCF [8]. Once CAS occurs, it leads to head and neck swelling, high venous pressures and resultant thrombosis with complex and difficult treatment options [8,9,10]. The arch is elastic, resistant to repeated angioplasty, and often requires stent placement resulting in further stenosis. A better understanding of the mechanisms leading to CAS is required in order to improve access procedures and maintenance.

CAS is believed to arise from hemodynamic alterations resulting from fistula creation. The etiology of CAS is complex and not well understood. However, there are several factors that suggest a strong hemodynamic role in development of CAS: 1) CAS occurs locally well downstream from the anastomosis, 2) the primary change in the cephalic arch after the fistula procedure is hemodynamic, and 3) there is a strong correlation between cephalic arch geometry and onset of stenosis. When a BCF is created, the anastomosis is between the brachial artery and the cephalic vein in the mid forearm. The creation of a fistula results in blood flow from an artery to a vein that is inherently non-physiologic in a number of ways. The initial flow rate in the radial artery of 20-30 mL/min increases to 200-300 mL/min immediately after creation of an AVF, reaching flow rates of 600-1200 mL/min after maturation [11]. In addition, the blood flow in the cephalic vein is not pulsatile prior to fistula insertion, whereas it is after the procedure. High fistula blood flow, a prerequisite for venous dilation and a requirement for easy cannulation and adequate dialysis, is accompanied by the high arterial pressure being transmitted to the vein. This intense increase in flow rate and pressure obviously has a profound effect on the hemodynamics in the downstream vein. [12]. For example, the flow through the cephalic vein after BCF creation may be transitional, or even turbulent, and altered shear stress related to the curve of the cephalic arch could lead to intimal injury and subsequent stenosis. The details of the hemodynamics throughout the cephalic arch is largely unknown.

Low wall shear stress (WSS) is thought to cause intimal hyperplasia (IH). The WSS in a blood vessel is influenced by the vessel geometry, the blood flow rate, and properties of the blood. In addition, hemodynamic forces play a critical role in the regulation of vessel diameter, which can change locally in order to maintain values of WSS within an acceptable range [10,13,14,15,16,17,18,19,20]. Although the dramatically increased blood flow at the time of fistula creation eventually leads to a corresponding increase in the overall shear stresses experienced by the cephalic vein [21], our computational model reveals that localized shear stresses in the cephalic arch can be lower than that experienced prior to fistula creation (see preliminary study). This is due to localized reversed flow conditions that can arise in curved vessels at high flow rates. As a result, regions of low shear stress may lead to the development of IH in order to adapt the vein such that the WSS returns to physiologically acceptable values [22]. We note, however, that the etiology of stenosis is primarily derived from arterial studies; therefore, it must be confirmed that the same hemodynamic factors apply in veins as well.

Dramatically altered hemodynamic conditions due to AVF can cause an abnormal response that may result in a pathological condition, such as CAS. There is likely a finite range of flow conditions for which the intimal adaptation process can accommodate and maintain stresses within their target ranges. The high flow rates that exist in the cephalic arch after fistula

creation can cause IH to lead to CAS, which further disturbs the flow [19,23,24]. As shown in our preliminary studies, as the stenosis worsens, high local shear stresses occur that can cause denudation of the endothelial layer, expose endothelial cells to activated platelets, and ultimately lead to thrombosis, causing the fistula to fail as a consequence of CAS [25]. Both the adaptation, through IH, and thrombosis responses are controlled to a large extent by shear stress levels. Therefore, extremely low and high WSS can have undesirable consequences, and both of these conditions may be present in the cephalic arch for BCF patients with CAS.

CAS may result from IH or defective remodeling of the vein. In addition to IH, CAS may also be caused by defective remodeling, which is characterized by a remodeling index (RI) [26,27]. Decreased WSS can result in IH [16], and venous wall hypertropic remodeling (increased wall thickness) is caused by increased circumferential stress due to higher blood pressures [20]. In an environment of reduced WSS and increased venous pressures, as is the case in the cephalic arch after fistula insertion, both adaptation via IH and remodeling may be present simultaneously. Therefore, in subjects with AVF, the resulting venous stenosis may be a combination of IH and wall hypertrophy induced remodeling.

Histology of the cephalic vein and other biological markers are important to consider as they also contribute to the development of intimal hyperplasia. The proposed investigation will provide insight into factors that contribute to the development of CAS in BCF. In addition to the hemodynamic factors highlighted above, other factors may also play a role. These include underlying histology of the cephalic vein, activation of numerous cytokines and growth factors, and generation of asymmetric dimethylarginine (ADMA), all of which may hasten the development of stenosis [19,20,28]. ADMA, a known inhibitor of nitric oxide, is a biomarker that may accumulate to high levels in patients with ESRD [29]. ADMA is known to participate in regulation of vascular tone and predisposes to the development of IH in transplant vasculopathy and coronary atherosclerotic states [30,31]. Alteration in this biomarker may contribute to the IH observed in patients with CAS. Studies on pre-bypass long saphenous veins suggest that the preexisting disease may contribute to the development of later stenosis [32]. In addition, little attention has been paid to the condition of the cephalic vein before creation of an AVF. The current proposal offers a perfect platform with which to collect data from biological markers and pathologic samples as outlined. The samples collected in this investigation will aide in the understanding of the poorly understood mechanisms of intimal hyperplasia and excessive remodeling in venous environments in a future proposal.

The angle of the cephalic arch, which tends to be wider in diabetics, strongly influences its hemodynamics. From our previous investigation [33], we find that the cephalic arch has an angle, α, that typically varies between 90 and 150 degrees, and this variability has important fluid dynamical and biological consequences. Our previous results have shown that diabetes is negatively correlated with CAS [8]. This is counterintuitive as diabetics are a subset of patients with worse overall access outcomes [34]. In order to determine if the cephalic arch geometry differs between diabetics and non-diabetics, we reviewed 57 venograms (12 had evidence of a stent and were eliminated from further analysis) of patients with BCF access and made the following measurements cephalic arch angle (α), which is a global measurement of the overall shape of the arch, minimum radius of curvature (R), which is a local measurement of the shape of the arch in the region where the arch changes most rapidly, and the cephalic vein diameter (d). Both global and local measurements show evidence of two distinct arch types. Patients having diabetes show a significant probability of having a larger R/d ratio and wider (more gradual) arch angle α than non-diabetics.

In addition, it has been observed in our preliminary study that the cephalic arch angle often changes, becoming more acute, over a period of months to years after creation of the fistula. This angle change is in addition to the increased radius of the vein immediately after the fistula is created. Angle change and radius increase both act to influence HDP. Therefore, not only does the cephalic arch geometry strongly influence the hemodynamics, the altered hemodynamics can lead to changes in the arch geometry.

Surgical construction of the anastomosis in a fistula and underlying systology and anatomy influences the hemodynamics in the cephalic arch. When creating a BCF, ideally the cephalic vein is anastomosed to the artery just distal to the antecubital crease. The proximal radial or brachial artery may be used as inflow with the anastomosis to the cephalic vein being 4--5 mm. The commonly constructed anastomosis is either end-to-side or side-to-side depending on the distance the vein is from the artery and the amount of mobilization required. An end-to-side anastomosis would be expected to lead to an initially higher flow rate in the cephalic vein and potentially worse endothelial damage as compared to a side-to-side anastomosis. The influence of the anastomotic length, distance from the cephalic arch, and type of construction have not been critically evaluated. A determination as to which of these factors contributes to CAS would aid in the construction of an anastomosis that would minimize the likelihood of CAS. Veins generally have valves for the purpose of preventing blood reflux. There are on average two valves in the cephalic arch in most individuals. Although in rare instances these venous valves have been implicated as potentially causing hypertrophy and lumen narrowing [35], it is our experience at the University of Chicago that as the cephalic vein dilates after creation of a BCF, the valves leading up to and in the arch become non-functioning and no longer influence the blood flow through the cephalic arch and therefore are not likely to be clinically significant.

Additional influences include the dynamic distensibility of the vein during the cardiac cycle and the occasional presence of aneurisms in the cephalic vein. The general consensus is that distensibility only has a secondary effect on HDP [16]. Therefore, it is not believed to be necessary to include for correlating HDP and the onset of CAS. Aneurisms proximal to the anastomosis and distal to the cephalic arch can influence the blood flow prior to reaching the arch. This will be accounted for by performing the Doppler measurements immediately distal to the cephalic arch.

Routine surveillance protocols to prevent fistula access complications are not available, but they would be expected to improve fistula outcomes if developed. While fistula access is recommended for all patients with ESRD on hemodialysis, complications, such as thrombosis, lead to access failure and poor outcomes. Therefore, surveillance of hemodialysis access is mandated by regulatory agencies [36]. Prior research attempts to substantiate a surveillance protocol for AV grafts has failed, showing no improvement in the outcome of thrombosis, and there is a paucity of data on the benefit of surveillance for fistulas [37]. The clinical advantage of early detection of venous stenosis including CAS is uncertain as there is not an acceptable surveillance method nor proven treatment options that prevent thrombosis and subsequent loss of access. Once the factors that cause CAS are known, however, early detection provides the necessary framework to develop protocols to mitigate the onset of CAS. Treatment trials in future studies could then be initiated that change Hct with dose adjustment of EPO, thereby altering the blood viscosity. In addition, one could change the anatomic location and/or type of anastomosis, decrease blood flow during dialysis, etc. The current proposal could serve as a platform to develop such surveillance protocols.

In summary, improved patency of the increasingly common AVF is necessary to provide better outcomes for hemodialysis patients. The primary failure mode of BCF is CAS, which may lead to thrombosis; therefore, the etiology of CAS is sought for BCF patients. The current treatment protocol for hemodialysis patients involves performing venograms only when clinically indicated because of head and neck swelling, high venous pressures and resultant thrombosis. When such venograms are obtained, CAS of 50% is considered clinically relevant, which when exceeded requires angioplasty, stent insertion, etc. in order to maintain access. This 50% stenosis threshold is a general criteria and does not take into account the non-physiologic hemodynamics within the curved cephalic arch with BCF.

This study represents a combined clinical and computational investigation in which computational fluid dynamics (CFD) is utilized to non-invasively determine the HDP. Development of this technical capability will ultimately revolutionize clinical practice by 1) determining whether a patient is a good candidate for a BCF, 2) improving BCF patency through revised clinical surveillance and treatment protocols, for example by determining patients who are more susceptible to CAS for whom additional surveillance would be required, 3) establish guidelines for changes in HDP that may portend CAS, 4) provide revised guidelines for what levels of CAS are clinically significant for a specific patient, 5) reduce the need for surgical interventions, and 6) decrease the overall cost of access.

The most important direct outcome of the investigation will be whether there is a correlation between HDP and CAS, and if so, what it is. If there is no such correlation, this will signal a complete reevaluation of the role of HDP in stenosis of veins as compared to arteries. More generally, the investigation will demonstrate how CFD can be incorporated into a clinical environment in which HDP are required for diagnosis, making treatment decisions, and/or predictive capability.

Purpose of the Study:

The long-term objective of the proposed investigation is to develop methods and procedures that will improve dialysis treatment by extending the long-term patency of brachiocephalic fistulae (BCF) and reducing the number of post-fistula procedures required to maintain such access. Not only will this lead to better patient outcomes, it is also likely to decrease the overall cost of maintaining access. This will be accomplished through an innovative combination of clinical and computational approaches seeking to test the hypotheses concerning the factors that lead to the development of local cephalic arch stenosis (CAS) and associated overall changes to the cephalic arch geometry in end stage renal disease (ESRD) patients with BCF.

If our hypotheses prove correct, the project outcomes will lead to 1) guidance for clinical management to minimize the risk of hemodynamic parameters (HDP) that cause CAS, 2) establishing a surveillance protocol to detect early signs of CAS or changes in HDP that would foster CAS, 3) suggesting alternative anastomosis designs for BCF that minimize risk of CAS, and 4) identification of patients for whom BCF would not be ideal. If our first hypothesis is incorrect, this will suggest that the HDP that lead to intimal hyperplasia and stenosis in arteries does not apply to veins, which have received very little attention. Therefore, a positive result will lead to significant improvements in clinical management of hemodialysis access, and in the unlikely event that HDP do not correlate with CAS, this will signal that the etiology of stenosis in veins will need to be investigated separately from that of arteries. As a result, all possible outcomes of the proposed investigation are clinically and/or scientifically important.

Methods:

Subject Population: Subjects for consideration in the study will be those referred from the out-patient nephrology clinic at the University of Chicago or a dialysis unit affiliated with DaVita Dialysis for a primary AVF. Eligible patients are those who are evaluated at the University of Chicago by a transplant or vascular surgeon. If the treating surgeon determines that the patient will have an attempted BCF, these patients will be eligible to participate in this study. The research team will be notified of a potential subject.

Clinical Protocol: After the patient is enrolled, pre-operative labs and work-up will be at the discretion of the surgeon. At the time of the OR, if a BCF is placed, labs will be drawn for viscosity, hematocrit, and ADMA. If it is determined at any time by the treating surgeon that a subject is not a suitable surgical candidate for a fistula due to underlying medical conditions, the subject will be withdrawn from the study. A venogram and Doppler of the cephalic arch will be obtained prior to anastomosis creation. A segment of the cephalic vein will be excised and sent to the Human Tissue Research Center to be preserved for future assay for histology, cytokines, and growth factors as discussed below. The serum specimens for viscosity, Hct, and ADMA will be transported on ice to IIT for assay. The patient will then have follow up Doppler, venogram, and associated tests according to the same procedure following the schedule provided in table \ref{protocol} for the large-scale prospective study. The clinically obtained measurements of geometry, flow rate, and whole blood viscosity will be used as input to the CFD model that will be used to compute the WSS and other HDP throughout the arch. Patients who develop symptoms of stenosis between protocol measurements will have a venogram performed at the time of diagnosis. These venograms will be considered standard of care, although data may be used for study analysis. In addition, excised tissue samples of the cephalic vein will be obtained when the access is placed and at each surgical revision and banked for future studies.

A subset of ten subjects from the large-scale study will be invited for a sub study that will involve: 1) more detailed three-dimensional imaging of the cephalic arch using IVUS, and 2) blood flow monitoring via Doppler during dialysis. Subjects who have not undergone previous dialysis treatment will be invited to participate in this optional sub-study. Participation in this sub-study will not affect participation in the main study. The IVUS imaging will provide a three-dimensional image of the vessel geometry and remodeling characteristics of the vein upon which the remodeling index (RI) will be computed [26,27]. Detailed CFD modeling will be carried out on these cephalic arch reconstructions, which will be performed at 0 and 24 months after fistula insertion. This will allow for a detailed prospective study of the evolution of IH, remodeling, and stenosis in the cephalic arch.

For the same ten subjects in the small-scale study, actual venous blood flow will be measured during hemodialysis treatment using a hand held Doppler device in the upper arm cephalic vein as close to the cephalic arch inlet as possible. The Doppler readings will be obtained at dialyzer blood flows of 300, 350, 400 and 450 mL/minute. Three measurements will be obtained at each

of four blood flows, and the average at each blood flow calculated. These readings will be taken at 0, 6, 12, 18, and 24 months after fistula insertion. The Doppler data obtained during hemodialysis will be used along with recent clinical venograms and laboratory data outlined above to perform CFD modeling. This data during dialysis treatment will be used to 1) quantify the differences in the HDP for subjects pre-dialysis and while on dialysis, and 2) seek a correlation between the Doppler readings at the various dialyzer flow rates and the onset of CAS. It is expected that formation of stenosis would alter the resistance to blood flow through the arch, thereby altering the flow rate in such a way that a unique signature will be apparent in the Doppler data during the controlled dialysis process. If this is the case, then additional surveillance techniques could be developed for identifying the onset of CAS.

History and Physical: The history and physical will be focused on the dialysis access. The history will include type and problems with cannulation, pain score with cannulation, needle size used, and bleeding complications. The physical exam will include an extensive examination of the access including pulse, description of anastomosis, any evidence of aneurisms, hematoma, inflammation, edema, or swelling of the head and neck.

Venogram: The venogram is performed as follows with simultaneous blood flow measurements. The patient will have their fistula punctured with a 21-gauge needle near the arterial anastomosis directed toward the stenosis (either venous or arterial). The needle will be exchanged for a 5 French dilator, and a digital subtraction venogram encompassing the outflow from puncture site to the right heart will be performed. Any stenosis will be measured using electronic calipers and defined. Any significant hemodynamic stenosis, defined as greater than 50% narrowing of the expected luminal diameter, will be treated with balloon angioplasty according to DOQI consensus from the International Society of Interventional Radiology [36]. In addition to the baseline venogram at the time of BCF placement, the venogram will include an image of the anastomosis and a measurement of the length from the anastomosis to the inlet of the cephalic arch.

Intravenous Ultrasound (IVUS): The subset of patients for the small-scale study will have a venogram performed as above and IVUS imaging in the Cardiac Catheterization Laboratory at the University of Chicago. The IVUS catheter will be inserted after the venogram, and images will be taken during pullback of the catheter at a set rate in order to provide cross-sectional images of the intimal and lumen layers of the vein at preset intervals along the cephalic arch [26,40].

Doppler: Doppler spectral analysis will be performed by an interventional radiologist at the time of each venogram or IVUS to measure velocity in the cephalic vein prior to entering the cephalic arch. The peak systolic velocity (PSV) at a 60-degree angle of insonation will be measured in the straight portion of the cephalic vein between the anastomosis and the arch as close to the arch as possible. The velocities in this location will be measured over several heart cycles and the average calculated.

Whole Blood Viscosity (WBV), Hematocrit, and ADMA: WBV, Hematocrit, and ADMA will be measured from serum samples obtained from patients pre-op or pre-dialysis. Blood samples will be anti-coagulated with 3.2% buffered sodium citrate and transported on ice to IIT. At IIT,

WBV will be measured using a Brookfield Programmable DV-II+ cone plate viscometer. ADMA will be measured using an ADMA ELISA kit from EUROIMU US or by HPLC. The samples will be collected every 6 months on all subjects and frozen to be run in aliquots of 50 at IIT.

Cephalic Vein Tissue Samples:
During placement of BCF vein tissue is excised during anastomosis to create the fistula. After the tissue is removed a small portion will be removed and collected for research from this discarded material. The cephalic vein samples will be collected and preserved in a tissue bank for later review by light and electron microscopy in a subsequent study. The sections will be reviewed by the pathologist for: generalized wall thickness, fibrous tissue infiltration, intimal hyperplasia, loss of endothelial cell layer, disruption of internal elastic lamina, mural calcification, and inflammatory reaction in the wall with infiltration by erythrocytes and/or histiocycyte. The changes that are present will be correlated with the development of intimal hyperplasia as evident by CAS. Our hypothesis is that histiopathic changes of intimal hyperplasia at baseline will correlate with the development of an accelerated course to develop CAS and subsequent thrombosis.

End-Point: The end-point of a subject's enrollment in the study will occur if they develop CAS of greater than 50%, transfer out of the DaVita dialysis program, transplantation, or death.

Special precautions to be taken by the researchers

The potential risks associated with the study include: venipuncture at the time of venogram, blood sample for labs (WBV, Hct and ADMA), intravenous contrast, venogram risks and IVUS risks.
The risk of venipuncture includes discomfort at the site of puncture; possible bruising and swelling around the puncture site; and rarely an infection. All anti-coagulants including: aspirin, NSAID, Plavix and Coumadin will be held for 3-7 days prior to the procedure to avoid bleeding complications.

The blood samples to be drawn (Hgb, ADMA and WBV) will require approximately 14 mL of blood. This is a minimal amount (less than 1 tablespoon) and should not affect the blood count.

The risk of contrast includes allergic reaction and renal failure in those patients who are not yet on hemodialysis. A minimal amount (50-100 cc) of non-ionic contrast media will be used. A careful history will be taken to determine past history of contrast allergy. If a contrast allergy history is obtained, the patient will be given pre-procedure steroids and Benadryl and non-ionic contrast will be used. If respiratory distress occurs as a result of contrast despite pre-medication with steroid and Benadryl, the subject will be withdrawn from the study. If the patient is not yet on hemodialysis, but has evidence of advanced renal failure, carbon dioxide gas will be used as a contrast agent, as there is no significant risk of renal failure. Physical risks include bleeding and infection.

The IVUS carries the rare possibility of: dissection of the wall of the vein, thrombosis, distal embolization with potential for limb loss, hematoma with potential nerve compression. We must

also include loss of the fistula as a potential complication. Of course, we don't expect any of these, but do mention these whenever we do invasive interventions in peripheral vasculature.

There are no physical risks involved with performing a venous Doppler.

The removal of a portion of the cephalic vein occurs when an anastomosis is created during a fistula creation. This excess tissue is usually discarded. However, from the subjects enrolled in the study a portion of the excess vein tissue will be collected after removal from the body and sent to the pathology lab for tissue banking for future studies. This will not add any additional time to the operative procedure and adds no significant risk to the operative procedure of fistula placement as it is standard practice to remove vein tissue which is discarded after the procedure.

Female subjects who are child bearing age will have a pregnancy test preformed prior to initial venograms, this is considered standard of care.

Description of experimental controls and use of placebos:

The following reviews the two aims with comparison, and statistical methods used in the current protocol. There will be no placebo used in this study.

Aim 1: Observe a cohort of BCF subjects over time with protocol venograms and Dopplers and calculate WSS, along with other HDP, using CFD in order to perform a life table analysis to estimate the effect of WSS on time to CAS. Because diabetes may affect this outcome, diabetic and non-diabetic patients will be studied. The large-scale prospective study will be augmented by a small-scale sub study involving a subset of the larger cohort. These patients will be subject to more detailed imaging and CFD in order to elucidate the mechanisms leading to IH and venous remodeling in the cephalic arch. The prospective design of the trial with arteriovenous fistulae (AVF) placement will allow cephalic vein tissue samples to be saved for pathologic review of histology and assay for cytokines and growth factors in a subsequent study.
Analysis 1: Primary Comparison 1: Risk of CAS in relation to minimum WSS magnitude.
Statistical Method: Analysis of time to CAS onset with Cox model that includes age, sex, diabetes status, end-to-side vs. side-to-side, WSS and other HDP. **Power Analysis:** We will fit a Cox regression model for time to event (CAS) using WSS at three months as covariate. For a change in WSS of one standard deviation (0.06 Pa according to our preliminary data), a sample size of 100 will allow us to detect a hazard ratio of 1.42 with 80% power at 5% two-sided significance level allowing for an R square of 0.10 (corresponding to a correlation of 0.32). An event rate of 72% is anticipated assuming a recruitment of 33 patients per year for three years, minimum follow up of 2 years, an exponential rate of occurrence of stenosis of 0.5 per year (based on our preliminary data) and an exponential loss rate (drop out and individuals dying prior to developing stenosis) of 1/10 per year. The sample size calculation is based on the estimated number of events. Other covariates such as diabetes, age, sex, type of anastomosis (side-to-side or end-to-side), and other HDP for potential effect on time to CAS. As a secondary analysis, we will use WSS as a time dependent variable.
Interpretation and Rationale 1: 1) WSS, or other HDP, predicts CAS. This supports our hypothesis. The hypothesis may hold for diabetics, non-diabetics, or both. Given this outcome, one could attempt alternative designs for BCF, or perhaps establish clinical management

protocols to minimize the effects of HDP that are shown to predict CAS. In addition, the results could be used to monitor patients in order to detect early signs of CAS or HDP that are changing in a manner that would foster CAS. Ultimately, we seek to identify the HDP that correlate with, and presumably cause, CAS. With this correlation, we can calculate the range of flow rates that will reduce the risk of CAS given a particular geometry as revealed by pre-operative venograms. **2) WSS, or other HDP, does not predict CAS.** This rejects our first hypothesis and would contradict the etiology of stenosis as determined primarily from arterial studies. This would also be an important scientific discovery that would alter present thinking about mechanisms for stenosis formation in veins.

Aim 2: In the same subjects as Aim 1, measure venous radius and cephalic arch angle over time and derive predictive functions of time, inlet pressure, anastomosis type, and initial arch angle.
Analysis 2: Primary Comparison 2: Correlation of maximum change in average venous radius and change in arch angle with type of fistula, initial arch angle, and inlet pressure at the time of fistula formation.
Statistical Method: Predictive models of venous radius and arch angle (outcome variables) using inlet pressure, initial angle, initial radius, time, and type of anastomosis as predictors will be developed. Linear and quadratic terms of time and other variables may be used. Splines basis may be used judiciously to improve model fit without over-fitting the data. The longitudinal nature of the data will be taken into account using an AR(1) correlation structure. We will assess the need to account for other covariates such as age, diabetes, and sex. Model selection will be performed using the approach described in Harrell (2001) [39]. **Power Analysis**: Assuming a simple linear regression model (only for power estimation) a sample size of 100 will allow us to identify the covariates as significant ($\alpha = 0.05$) with 80% power if indeed they jointly explain at least 12% of the radius' variability.
Interpretation and Rationale 2: 1) Radius/angle correlates with pressure, anastomosis type, and/or initial angle. We will use the mean change and SD in our predictive model. Geometry (radius and angle) is crucial for our computational model. A larger radius allows for a lower inflow velocity for any given flow rate produced by the anastomosis. The venograms give us the starting radius, but that radius will not be present during the life of the BCF. The starting radius is augmented by the predicted increment due to arterialization. We will derive a predictive formula for this increment in radius from the observations in this aim and incorporate it into our computational model. More acute angulation reduces WSS locally. We have evidence that the angle changes over time from that present at surgery. Therefore, if we can predict the change in angle with time from this aim, we can refine our estimates of WSS and other HDP over the life of the BCF. **2) Radius/angle does not correlate with pressure, or angle, or type.** This effect will not be incorporated into the predictive model

<u>Exact location where research is to be conducted</u>:

Patients will be recruited primarily at the out-patient clinic located at the DCAM, University of Chicago as they present to have an evaluation for a permanent access. If is determined that a BCF is the optimal access, they will be referred for screening to an intake coordinator who is IRB approved for the study.

Patients will also be recruited from Davita dialysis units affiliated with the University of Chicago and others units of Davita dialysis as needed. Patients will be identified as needing permanent access and be referred for screening to an intake coordinator who is IRB approved for the study.

Pre-procedure labs include a PT/INR and Platelet will be collected within one month of the radiology procedure.

All labs (Hct, ADMA and WBV) will be collected pre-procedure for the venograms and transported to IIT for analysis.

Duration of protocol: 5 years

Number of experimental subjects, inclusion and exclusion criteria:

The expected number of subjects for enrollment is 100. 33 to 34 patients per year will be recruited for a total of 100. Patients will be recruited for the first three years of the study and followed up through the end of the study (at least 2 years).

Subjects:
Inclusion Criteria:
1. Subjects enrolled in the study will include patients with irreversible chronic renal failure; either those receiving chronic hemodialysis or anticipation that hemodialysis will be required.
2. Subjects for consideration in the study will be referred from the out-patient nephrology clinic at the University of Chicago or a dialysis unit affiliated with DaVita Dialysis for a primary AVF. The patient will be referred for AVF placement and evaluated at the University of Chicago by a transplant or vascular surgeon. If it is determined that the patient is a candidate for fistula placement, the surgeon will decide based on physical exam, the best location for the access. If the optimal location is a proposed BCF, a research coordinator will be notified and the patient will be enrolled by written consent approved by the IRB from the University of Chicago.
3. Subjects who can provide consent or consent of a surrogate through proxy

There will be no exclusion based on sex, race or ethnic group. However, because of the geographic location of the University of Chicago, we anticipate that a large proportion of patients enrolled in the study will be of African American origin.

Exclusion Criteria:
1. Patients will be excluded if they are less than 21 years of age
2. Pregnant females will be excluded. A careful history will be taken and women who are pregnant or have a clinical indication will have a serum pregnancy test with a positive result will be excluded.
3. Those with a known history of anaphylaxis from contrast
4. Those who are found to be ineligible to have a surgical placement of a BCF as determined by the surgeon

The research topic to be studied is not relevant to children.

Sub-study
We plan to enroll 10 subjects in this addition observation. Enrollment in this sub-study will begin in the second year of the study life. Subjects will have the option to participate in this study which will not affect their participate in the main study if they meet the following criteria:

Inclusion Criteria:
1. No history of hemodialysis prior to BCF access placement

Exclusion Criteria:
1. History of hemodialysis prior to BCF access placement

Potential risks and benefits to subjects:

Potential Risks:
The potential risks associated with the study include: venipuncture at the time of venogram and instrumentation at the fistula includes rare possibility of: dissection of the wall of the graft, thrombosis, distal embolization with potential for limb loss, bleeding, hematoma with potential nerve compression. We must also include loss of the fistula as a potential complication. Of course, we don't expect any of these, but do mention these whenever we do invasive interventions in peripheral vasculature, and intravenous contrast. The risk of venipuncture includes discomfort at the site of puncture; possible bruising and swelling around the puncture site; and rarely an infection.
All anti-coagulants including: aspirin, NSAID, Plavix and Coumadin will be held for 3-7 days prior to the procedure to avoid bleeding complications. The risk of contrast includes allergic reaction and renal failure in those patients who are not yet on hemodialysis. A minimal amount (50-100 cc) of non-ionic contrast media will be used. A careful history will be taken to determine past history of contrast allergy. If a contrast allergy history is obtained, the patient will be given pre-procedure steroids and Benadryl and non-ionic contrast will be used. Pre-medication with Benadryl and steroids is standard of care pre-procedure for patients who have a known allergy to contrast. If a patient receives contrast and develops respiratory compromise from an allergic reaction despite pre-medication with steroids and Benadryl, the patient will be withdrawn from the study. If the patient is not yet on hemodialysis, but has evidence of advanced renal failure, carbon dioxide gas will be used as a contrast agent, as there is no significant risk of renal failure. The risk of blood loss with the blood draw is minimal as 14 mL is less than one tablespoon and there is no significant risk to this amount of blood loss. There are no physical risks involved with performing a venous Doppler.
Removal of the cephalic vein tissues at time of fistula placement adds no significant risk to the operative procedure of fistula placement and will not increase the length of the surgical procedure. Only leftover vein tissue which is discarded after the procedure will be used for research purposes.

There is a direct benefit as the patients enrolled will have a detailed work-up and follow through of their vascular access that will prevent complications. Standardized pre-op work-up and surveillance of access is currently not uniformly performed. This study will also lead to an

improved understanding of the effects of hemodynamics on vascular access and the development of stenosis.

The indirect benefits are an increased understanding of the pathogenesis of the mechanism of venous stenosis. This knowledge will improve our understanding of access failure, improving outcomes for patients with ESRD on hemodialysis and decrease morbidity and mortality in this patient population.

Payment to subjects:

There will be no direct payment to the subject although if enrolled the venograms/Doppler exams will be covered by the study unless they are required for standard of care.

Recruitment Methods:

University of Chicago access surgeons, research nurse coordinator and surgical nurse practitioner, from the University of Chicago will be informed of the study and asked to screen patients for participation.

Informed consent:

Informed consent will be obtained at the time of enrollment in the out-patient clinic or the dialysis unit. The out-patient clinic is located in the DCAM at the University of Chicago. The out-patient dialysis units include any affiliated DaVita unit. Informed consent will be obtained by the primary investigator or a co-investigator. Patients will be asked to read the specific terms detailed in this form and initial appropriate sections. They will be asked to sign the form including their agreement to participate. A copy of the form will be provided to the subject for their record.

Confidentiality:

In order to avoid this risk, each subject will be assigned a unique ID number so that anonymity is preserved according to HIPPA regulation. Only principle or co-investigators approved on the IRB will be involved in patient recruitment and providing clinical information needed for the analysis of the patients. Only they will have access to the file that links the patient’s history to the study ID number. Any documents containing patient identifiers will be kept in locked files in the PI’s office. Computer documents with patient data will be protected by passwords only known by the PI or the research team.

Data Monitoring Plan:

A template for data collection using excel will be developed for each patient. The research nurse for the study or the PI will be physically present during the consent, operative procedure and when venograms are preformed to ensure that the data is collected accurately. The operative

data will be reviewed with the surgeon of record. All data will be entered in the excel template within 24 hours and reviewed with the PI for accuracy at a weekly meeting with the PI.

Description of how the subject's primary physician will be notified of and, as appropriate, involved in the proposed research:

If a subject is identified as a candidate for the study, the primary care physician will be notified and informed of risks and benefits of the study.

Description of anticipated coordination between appropriate interdepartmental faculty, and where necessary, inclusion of those faculty as participants:

The study will have a full-time research nurse designated to assist with coordination between appropriate interdepartmental faculty at the University of Chicago and between IIT and U of C. There will be a weekly meetings between personnel form U of C and IIT to ensure the data is collected and analyzed accurately. A meeting will be a monthly meeting or more often as needed with IIT and U of C (PI, research nurse and interventional radiology) to ensure data is performed and collected accurately.

References:

[1]. United States Renal Database System (2009). http://www.usrds.org/.

[2]. Huang, E.S., Basu, A., O'Grady, M., & Capretta, J.C. Projecting the future diabetes population size and related costs for the U.S. *Diabetes Care* (2009) **32**: 2225--2229.

[3]. US Department of Health and Human Services (DHHS): ``Healthy People 2020" Chronic Kidney Disease: CKD HP2020–4.

[4]. Besrab, A., Work, J., Brouwer, D., et al. Clinical practice guidelines for vascular access. *Am J Kidney Dis* (2006) **48** (Suppl 1): S176--S247.

[5]. Miller, P.E., Tulwani, A., Lusey, C.P., et al. Predictors of adequacy of arteriovenous fistulas in hemodialysis patients. *Kidney Inter* (1999) **56**: 275--86.

[6]. Rodriguez, J.A., Armandans, L., Ferrer, E., Olmos, A., Cordina, S., Bartolome, J., Borrelas, J., & Piers, L. The function of permanent vascular access. *Nephrol Dial Transplant* (2000) **15**: 402--408.

[7]. Rajan, D.K., Clark, T.W., Patel, N.K., Stavropoulos, S.W., & Simons, M.E. Prevalence and treatment of cephalic arch stenosis in dysfunctional autogenous hemodialysis fistulas. J *Vasc Interv Radiol* (2003) **14**: 567--573.

[8]. Hammes, M., Funaki, B., & Coe, F.L. Cephalic arch stenosis in patients with fistula access for hemodialysis: relationship to diabetes and thrombosis. *Hemodialysis International* (2008) **12**: 85--89.

[9]. Klan, K. & Asif, A. Cephalic Arch Stenosis. *Seminars in Dialysis* (2008) **21**: 78--82.

[10]. Van Tricht, I., De Wachter, D., Tordoir, J., & Verdonck, P.} Hemodynamics and complications encountered with arteriovenous fistulas and grafts as vascular access for hemodialysis: A review. *Annals of Biomedical Engineering* (2005) **33**: 1142--1157.

[11]. Wedgewood, K.R., Wiggins, P.A., & Guillou, P.J. A prospective study of end-to-side vs.\ side-to-side arteriovenous fistulas for hemodialysis. *Br J Surg* (1984) **71**: 640--642.

[12]. Albayrak, R., Yuksel, S., Colbay, M., Degirmenci, B., Acarturk, G., Haktanir, A., & Karaman, O. Hemodynamic changes in the cephalic vein of patients with hemodialysis arteriovenous fistula. *J Clinical Ultrasound* (2006) **35**: 133--137.

[13]. Bassiouny, H., White, S., Glagov, S., et al. Anastomotic intimal hyperplasia: mechanical injury or flow induced. *Journal of Vascular Surgery* (1992) **15**: 708--717.

[14]. Binns, R., Ku, D. Stewart, M., et al. Optimal graft diameter: effect of wall shear stress on vascular healing. *Journal of Vascular Surgery* (1989) **10**: 326--337.

[15]. Glagov, S. Intimal hyperplasia, vascular modeling, and the restenosis problem. *Circulation* (1994) **89**: 2888--2891.

[16]. Ku, D.N. Blood flow in arteries. *Annual Review of Fluid Mechanics* (1997) **29**: 399--434.

[17]. Mattsson, E.J.R., Kohler, T.R., Vergel, S.M. & Clowes, A.W. Increased blood flow induces regression of intimal hyperplasia. *Arteriosclerosis, Thrombosis, and Vascular Biology* (1997) **17**: 2245--2249.

[18]. Nerem, R.M. Hemodynamics and the vascular endothelium. *Journal of Biomechanical Engineering* (1993) **115**: 510--514.

[19]. Roy-Chaudhury, P., Sukhatme, V.P., & Cheung, A.K. Hemodialysis vascular access dysfunction: a cellular and molecular viewpoint. *Journal of the American Society of Nephrology* (2006) **17**: 1112--1127.

[20]. Wootton, D.M. & Ku, D.N. Fluid mechanics of vascular systems, diseases, and thrombosis. *Annual Review of Biomedical Engineering* (1999) **1**: 299--329.

[21]. Remuzzi, A., Ene-lordache, B., Moscomi, L., et al. Radial artery wall shear stress evaluation in patients with arteriovenous fistula hemodialysis access. *Biorheology* (2003) **40**: 423--430.

[22]. Krishnamoorthy, M.K., Banarjee, R.K., Wang, Y., Zhang, J., Roy, A.S., Khoury, S.F., Arend, L.J., Rudich, S., & Roy-Chaudhury, P. Hemodynamic wall shear stress profiles influence

the magnitude and pattern of stenosis in a pig AV fistula. *Kidney International* (2008) **74**: 1410--1419.

[23]. Corpataux, J.M., Haesler, E., Silacci, P., Ris, H.B., & Hayoz, D. Low-pressure environment and remodeling of the forearm vein in Brescia-Cimino hemodialysis access. *Nephrol Dial Transplant* (2002) **17**: 1057--1062.

[24]. Berger, S.A. & Jou, L.D. Flows in stenotic vessels. *Annual Review of Fluid Mechanics* (2000) **32**: 347--382.

[25]. Slack, S.M. & Turitto, V.T. Fluid dynamic and hemorheologic considerations. *Cardiovascular Pathology* (1993) **3**: S11--S21.

[26]. Koskinas, K.C., Feldman, C.L., Chatzizisis, Y.S., Coskun, A.U., Jonas, M., Maynard, C., Baker, A.B., Papafaklis, M.I., Edelman, E.R., & Stone, P.H. Natural history of experimental coronary atherosclerosis and acular remodeling in relation to endothelial shear stress. *Circulation* (2010) **121**: 2092--2101.

[27]. Von Birgelen, C., Hartmann, M., Mintz, G.S. Bose, D., Eggebrecht, H., Neumann, T., Gossl, M., Wieneke, H., Schmermund, A., Stoel, M.G., Verhorst, P.M.J., & Erbel, R. Remodeling index compared to actual vascular remodeling in atherosclerotic left main coronary arteries as assessed with long-term (> 12 months) serial intravascular ultrasound. *Journal of the American College of Cardiology* (2006) **47**: 1363--1368.

[28]. Wali, M.A., Eid, R.A., Dewan, M., & Al-Homray, M.A. Intimal changes in the cephalic vein of renal failure patients before arteriovenous fistula (AVF) construction. *J Smooth Muscle Res* (2003) **39**: 95--105.

[29]. Zoccali, C. Asymmetric dimethylarginine (ADMA): a cardiovascular and renal risk facto on the move. *Journal of Hypertension* (2006) **24**: 611--619.

[30]. Aslam, S., Santa, T., Leone, A., & Wilcox, C. Effects of amlodipine and valsartan on oxidative stess and plasma methylarginines in end-stage renal disease patients on hemodialysis. *Kidney International* (2006) **70**: 2109--2115.

[31]. Tanaka, M., Sydow, K., Gunawan, F., Jacobi, J., Tsao, P., Robbins, R.C., & Cooke, J.P. Dimethylarginine dimethylaminohydrolase overexpression suppresses graft coronary artery disease. *Circulation* (2005) **112**: 1549--1556.

[32]. Batayias, G.E., Barboriak, J.J., Korns, M.E., & Pintar, K. The spectrum of pathologic changes in aortocoronary sapheous vein grafts. *Circulation* (1977) **56** (3 Suppl): II18--22.

[33]. Hammes, M.S., Boghosian, M.E., Cassel, K.W., Funaki, B., & Coe, F.L. Characteristic differences in cephalic arch geometry for diabetic and non-diabetic ESRD patients. *Nephrol Dial Transplant* (2009) **24 (7)**: 2190--2194.

[34]. Sedlacek, M., Teodorescu, V., Falk, et al. Hemodialysis access placement with perioperative noninvasive vascular mapping: comparison between patients with and without diabetes. *Am J Kidney Dis* (2001) **38**: 560--564.

[35]. Glanz, S., Bashist, B., Gordon, D.H., Butt, K., & Adamsons, R. Angiography of upper extremity access fistulas for dialysis. *Radiology* (1982) **142**: 43--52.

[36]. NKF: K/DOQI Clinical Practice Guidelines for Hemodialysis Adequacy, Update 2006. *American Journal of Kidney Diseases* (2006) **48**: S2--S90.

[37]. Allon, M. & Robbin, M.L. Hemodialysis vascular access monitoring: Current concepts. *Hemodialysis International* (2009) **13**: 153--162.

[38]. Ward, M.R., Pasterkamp, G., Yeung, A.C., & Borst, C. Arterial remodeling: Mechanisms and clinical implications. *Circulation* (2000) **102**: 1186--1191.

[39]. Harrell, F.E. Regression modeling strategies with application to linear models, logistic regression, and survival analysis. (2001) Springer, New York.

[40]. Coskun, A.U., Yeghiazarians, Y., Kinlay, S., Clark, M.E., Ilegbusi, O.J., Wahle, A., Sonka, M., Popma, J.J., Kuntz, R.E., Feldman, C.L., & Stone, P.H. Reproducibility of coronary lumen, plaque, and vessel wall reconstruction and of endothelial shear stress measurements in vivo in humans. *Catheterization and Cardiovascular Interventions* (2003) **60**: 67--78.

[41]. Lee, S., Piersol, N., Kalata, W., Skelly, C.L., Curi, M.A., Fischer, P.F., & Schwartz, L.B. Numerical Simulation of vein graft hemodynamics. *Bioengineering Conference,* (2001): 50.

[42]. Loth, F., Fischer, P.F., Arslan, N., Bertram, C.D., Lee, S.E., Royston, T.J., Shallan, W.E., & Bassiouny, H.S. Transitional flow at the venous anastomosis of an arteriovenous graft: potential activation of ERK1/2 mechanotransduction. *Journal of Biomechanical Engineering* (2003) **125**: 49--61.

[43]. McMillan, D.E. Disturbance of serum viscosity in diabetes mellitus. *Journal of Clinical Investigation* (1974) **53**: 1071--1079.

[44]. Chen, J., Chang, K., Lin, K., Lee, J., & Huang, S. Ultrasonographic predictors of unsuccessful cephalic vein approach during pacemaker or defibrillator lead implantation. *PACE* (2006) **29**: 706--711.

B – Study schema

	Month after Fistula Insertion													
	Baseline	1	3	6	12	18	24	30	36	42	48	54	60	Access Failure
History	X	X	X	X	X	X	X	X	X	X	X	X	X	
Physical	X	X	X	X	X	X	X	X	X	X	X	X	X	
Venogram	X		X		X		X		X					X
Doppler	X		X		X		X		X					
Viscosity	X		X		X		X		X					
Hct	X		X		X		X		X					
ADMA	X		X		X		X		X					
Collection of excised vein tissue	X													X[4]
PT/INR[2]			X		X		X		X					
**Sub-Study[1]**														
IVUS [3]					X				X					
Handheld Doppler during dialysis		X		X	X	X	X							

1 Applies select sub-set of 10 subjects
2 If not drawn within a month of venogram as standard of care
3 IVUS will replace venogram and doppler at these time points for sub-set 10 subjects
4 At the time of surgical resection if clinically necessary for CAS a sample will be collected and stored
